# Overuse in cancer care: do European studies provide information useful to support policies?

**DOI:** 10.1186/s12961-018-0287-z

**Published:** 2018-02-20

**Authors:** Roberto Grilli, Valentina Chiesa

**Affiliations:** 1 Clinical Governance Program, Local Health Authority – IRCCS of Reggio Emilia, Reggio Emilia, Italy; 2 Department of Medicine and Surgery, Unit of Biomedical, Biotechnological and Translational Science, University of Parma, Parma, Italy

**Keywords:** Quality of care, Cancer, Low value care, Overuse, Underuse, Review

## Abstract

Health services overuse has been acknowledged as a relevant policy issue. In this study, we assessed the informative value of research on the quality of cancer care, exploring to what extent it is actually concerned with care overuse, thus providing policy-makers with sound estimates of overuse prevalence. We searched Medline for European studies, reporting information on the rate of use of diagnostic or therapeutic procedures/interventions in breast, colorectal, lung and prostate cancer patients, published in English between 2006 and 2016. Individual studies were classified with regards to their orientation towards overuse according to the quality metrics adopted in assessing rates of use of procedures and interventions.

Out of 1882 papers identified, 100 accounting for 94 studies met our eligibility criteria, most of them on breast (*n* = 38) and colorectal (*n* = 30) cancer. Of these, 46 (49%) studies relied on process indicators allowing a direct measure of under- or overuse, the latter being addressed in 22 (24%) studies. Search for overuse in patterns of care did not increase over time, with overuse being measured in 24% of the studies published before 2010, and in only 13% of those published in 2015–2016. Information on its prevalence was available only for a relatively limited number of procedures/interventions. Overall, estimates of overuse tended to be higher for diagnostic procedures (median prevalence across all studies, 24%) than for drugs, surgical procedures or radiotherapy (median overuse prevalence always lower than 10%). Despite its increasing policy relevance, overuse is still an often overlooked issue in current European research on the quality of care for cancer patients.

**Electronic supplementary material:**

The online version of this article (10.1186/s12961-018-0287-z) contains supplementary material, which is available to authorized users.

## Background

A widespread concern is rising regarding the amount of resources wasted in healthcare delivery through the provision of low-value care, namely of interventions and procedures of little (if any) clinical value, or through the inappropriate use of otherwise effective healthcare interventions [1]. Overuse, defined as the provision of services that are more likely to cause harm than good [2], has been identified as a major policy issue, both for its clinical and economic implications [3]. Indeed, resources currently wasted in the delivery of unnecessary care could be reallocated to address health services underutilisation whenever it occurs, improving patient access to interventions known to be effective and clinically valuable.

However, tackling the issue of health services overuse is a hard challenge to policy-makers, calling for the development of coherent policy efforts able to identify and prioritise areas of inappropriate care, and develop coherent strategies to achieve the required changes in clinical practice. Therefore, the availability of good quality information on the actual prevalence of health services overuse in clinical practice is of utmost importance to inform this policy process.

The need to address the issue of overuse in healthcare delivery in order to improve the quality and efficiency of resource use is also of relevance in cancer care [4], where the provision of good quality care is a major economic challenge to healthcare systems, not immune from the problem of inappropriateness [5–7].

In the framework of the Joint Action on Cancer Control (CanCon) [8], a policy paper on the issue of health services overuse in cancer care has been written [4]. In the context of the development process, an analysis of studies on patterns of care for cancer patients in European countries was undertaken herein to assess their informative value for policies concerning the issue of overuse. In particular, beyond assessing for which procedures/interventions there is available information on overuse, we explored how utilisation rates were actually measured, as we consider that the type of quality metric adopted reveals the extent to which assessment of overuse is a real concern in this research area.

## Methods

Our main goal was to explore the degree of attention given to the search for overuse in the delivery of care to cancer patients through an analysis of the measures employed in assessing processes of care. We relied on the distinction between direct and indirect measures of process of care [9]. Indirect measures are simple rates, documenting the frequency of use of specific interventions/procedures in a population. Therefore, they can provide only indirect information on the existence of health services under- or overuse in clinical practice, in particular comparing rates across different providers or geographic areas. In these circumstances, empirical documentation of substantial variability is often taken as indirect evidence of under- or overuse, whenever rates are shown to be lower or higher than the average to a statistically significant extent [10]. While useful to highlight possible problems in patterns of care, interpretation of variation analysis can be complex, and the assumption that inappropriate use in excess (i.e. overuse) is higher where rates are also higher has never been so clearly demonstrated [11].

Direct measures are those allowing the opportunity to make a direct judgement about the clinical processes, as for the presence of under- or over-utilisation of health services. As such, they do not rely on assumptions, rather on the validity of the criteria used to distinguish between appropriate and inappropriate care. The availability of explicit eligibility criteria along with information on whether a procedure has actually been delivered, allow an appropriateness analysis able to provide estimates of both under- and overuse, if they exist, as outlined in Box 1. According to this schematic representation, prevalence of overuse can be made measurable relying on two different rates (C/A+C and C/C+D), having as denominator either the total number of patients exposed to a procedure or the total number of non-eligible patients. Therefore, overuse can be assessed from two different and complementary perspectives [12], respectively centred on procedures and patients. Further, the extent to which research on patterns of care is actually oriented towards the search for overuse is made evident by the type of measures (i.e. rates) adopted

**Table:** **Box 1** Schematic representation of different possible rates of under- and overuse of procedures/interventions

	Procedure/intervention delivered		Rates	Questions addressed by the rates
Patient eligible to the procedure/intervention	Yes	No		
Yes	A	B	B/A+B	How frequently did eligible patients fail to receive appropriate care? UNDERUSE
No	C	D	C/C+D	How frequently were non-eligible patients exposed to the procedure? OVERUSE
Rates	C/A+C	B/B+D		
Question addressed by the rates	How frequently has the procedure been delivered to non-eligible patients?	How frequently should patients not exposed to the procedure have received it?		
	OVERUSE	UNDERUSE		

### Identification of relevant studies

We undertook a literature search using Medline to retrieve articles (in English) from European studies providing information on the rate of use of diagnostic or therapeutic interventions, procedures and services in cancer patients. The search strategy was based on the presence in the title or abstract of words referring to ‘appropriateness’, ‘underuse’, ‘overuse’, and/or ‘quality of care’. As we anticipated that the quantity of studies was likely to be large, to keep the task more manageable, we focused on those concerning patients with breast, colorectal, lung and prostate cancer, and restricted the time frame of our search to the period January 2006 to June 2016, as older papers were likely to describe patterns of care no longer fully representative of current clinical practice. Details of the search strategy adopted are outlined in Additional file 1. In addition, the reference list of relevant available reviews [13–16] was inspected to identify any additional potentially relevant papers.

Experimental or observational studies aimed at assessing effectiveness or cost-effectiveness of healthcare interventions were excluded, as well as methodological papers and studies assessing the impact of quality improvement interventions. When studies describing quality of care were identified, they were excluded if concerning patterns of care provided by a single provider/centre (as their findings would have not been sufficiently generalisable), or when reporting only outcome indicators (i.e. mortality rates, complications rates, etc.) or self-reported information on patterns of care (i.e. through questionnaires targeted to health professionals or patients).

Titles and abstracts were independently reviewed for eligibility by the two authors (RG and VC) after piloting inclusion and exclusion criteria on a sample of 100 references, with satisfactory reliability (k = 0.70). Full texts were further examined when the abstract was insufficient or unclear. Disagreements were rare and resolved by consensus.

### Data abstraction

From individual papers meeting the inclusion criteria, the two authors gathered information regarding participating country, year(s) of care delivery, design (observational vs. cohort study, with the latter further distinguished into prospective and retrospective, according to patient recruitment and data collection), sample size, type of procedures/interventions considered, use of explicit standards (i.e. guideline recommendations), source of data and main findings. Further, relying on the analytic framework described above (Box 1), studies were classified into those (1) providing generic rates of use, when measures provided information on the frequency of utilisation of procedures/interventions, but without explicitly assessing the degree of compliance with local/regional/national guidelines, or with pre-defined appropriateness criteria; (2) providing rates oriented towards underuse, when the process indicators adopted were based upon utilisation rates of the type oriented towards measuring underuse (Box 1), relying on explicit criteria; and (3) providing rates oriented towards overuse, when at least one of the indicators adopted was based upon utilisation rates of the type oriented towards measuring overuse (Box 1), relying on explicit criteria.

Findings of individual studies were classified according to whether over- or underuse was explicitly referred to by authors, searching if the abstract or other sections of the papers (results, discussion, conclusions) reported some keywords (‘underuse’, ‘underutilisation’, ‘overuse’, ‘overutilisation’) or, alternatively, for sentences explicitly mentioning that some patient categories had not received (or inappropriately received) a specific intervention/procedure. Whenever overuse was detected according to the criteria outlined above, we collected detailed information on the estimated prevalence (i.e. numerator and denominator of the rate) if explicitly reported in the text or tables.

## Results

**Fig. 1:**
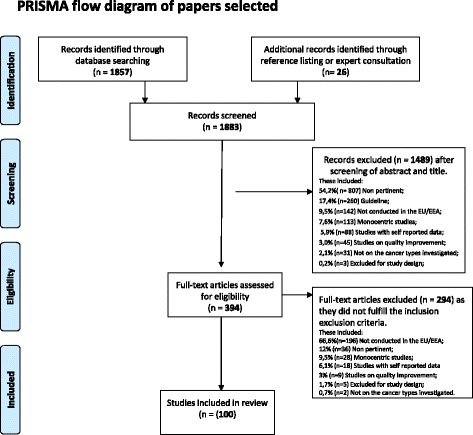
Flow chart of the selection process of papers relevant to the review

The flow chart of the search process is shown in Fig. 1. Overall, out of the 1833 papers originally identified, 100, accounting for 94 studies, met our eligibility criteria (Additional file 2). Of these, 38 were on breast cancer, 30 on colorectal cancer, and 8 and 9 on lung and prostate cancer, respectively. Nine studies included more than one of those tumours.

The general characteristics of the 94 studies identified are reported in Table 1. Only 7 (8%) had been conducted on patients from more than one European country. Overall, the Netherlands and the United Kingdom were the countries more frequently represented (in 21 and 19 studies, respectively), followed by Italy (*n* = 16), Germany (*n* = 14) and Sweden (*n* = 8).

**Table 1:** Characteristics of the 94 European studies on patterns of care for breast, colorectal, lung and prostate cancer, published between 2006 and 2016

	Breast cancer	Colorectal cancer	Lung cancer	Prostate cancer	Miscellaneous^a^	Total
	N (%)	N (%)	N (%)	N (%)	N (%)	N (%)
Year of publication						
≤ 2010 (%)	11 (29) (38)	7 (23) (24)	3 (38) (10)	4 (44) (14)	4 (44) (14)	29 (31) (100)
2011–2012 (%)	11 (29) (44)	8 (27) (32)	3 (38) (12)	1 (11) (4)	2 (22) (8)	25 (26.5) (100)
2013–2014 (%)	6 (16) (24)	13 (43) (52)	2 (25) (8)	1 (11) (4)	3 (33) (12)	25 (26.5) (100)
2015–2016 (%)	10 (26) (66.7)	2 (7) (13.3)	0 (0) (0)	3 (33) (20)	0 (0) (0)	15 (16) (100)
Study design						
Cross-sectional (%)	21 (55.3) (49)	8 (27) (19)	4 (50) (9)	4 (44) (9)	6 (67) (14)	43 (46) (100)
Cohort (%)	17 (44.7) (33)	22 (73) (43)	4 (50) (8)	5 (56) (10)	3 (33) (6)	51 (54) (100)
Data sources						
Cancer registry (%)	17 (44.7) (34.7)	18 (60) (36.7)	6 (75) (12.2)	7 (77.8) (14.2)	1 (11.1) (2)	49 (52) (100)
Cancer registry + administrative databases (%)	6 (15.8) (75)	1 (3.3) (12.5)	1 (12.5) (12.5)	0 (0) (0)	0 (0) (0)	8 (8) (100)
Administrative databases alone (%)	8 (21) (66.7)	1 (3.3) (8.3)	0 (0) (0)	1 (11.1) (8.3)	2 (22.2) (16.7)	12 (13) (100)
Other sources (%)	7 (18.4) (28)	10 (33.3) (40)	1 (12.5) (4)	1 (11.1) (4)	6 (66.7) (24)	25 (27) (100)
Type of measures						
Generic (%)	17 (44.7) (3.,4)	16 (53.4) (33.3)	5 (62.5) (10.4)	5 (55.5) (10.4)	5 (55.5) (10.4)	48 (51) (100)
Oriented towards underuse (%)	8 (21) (33.5)	10 (33.3) (42)	3 (37.5) (12.5)	1 (11.1) (4)	2 (22.2) (8)	24 (26) (100)
Oriented towards overuse (%)	13 (34.2) (59.1)	4 (13.3) (18.2)	0 (0) (0)	3 (33.3) (13.6)	2 (22.2) (9.1)	22 (23) (100)
Total (%)	38 (100) (40)	30 (100) (32.5)	8 (100) (8.5)	9 (100) (9.5)	9 (100) (9.5)	94 (100) (100)

^a^Studies including different types of cancers are in this category

In 46 (49%) studies, patterns of care were analysed through process indicators directly measuring degree of compliance with recommendations from practice guidelines or with consensus panel criteria. Out of those, 24 (25%) measured whether eligible patients received the recommended procedure/intervention and were therefore oriented towards assessing degree of underuse. Only 22 (23%) studies [17–42] adopted at least one process indicator directly measuring overuse, most of which (*n* = 13) dealt with breast cancer (Table 1). None of the lung cancer studies relied on overuse measures.

As shown in Table 2, measures of overuse were adopted to assess diagnostic tests [27, 28, 35, 36, 40], surgical procedures [17, 21–25, 29–31, 33, 38, 40], radiotherapy [17, 26, 29–31, 37, 38, 40], and drugs; this last category included studies measuring overuse of chemotherapies [17, 18, 20, 26, 29–31, 36, 42], hormone therapies [20, 26, 32, 39], trastuzumab [19], bevacizumab [34, 42], or erythropoiesis-stimulating agents [41]. Overall, as shown in Tables 3 and 4, the median value of overuse rates tended to be higher for diagnostic procedures, while median values for drugs, surgery and radiotherapy were always lower than 10%. That held true also when only the subgroup of studies on breast cancer was considered. For all the types of procedures and interventions, prevalence of overuse was highly variable across individual studies.

**Table 2:** Prevalence of overuse as reported in European studies on patterns of care for breast, colorectal and prostate cancer, published between 2006 and 2016

*Breast cancer*	*Country*	*Years of care delivery*	*Interventions/procedures*	*Prevalence of overuse (95% CI)*
Wockel, 2010 [17]	Germany	2001–2005	BCS in tumour size > 4 cm, in multicentric cancer, in inflammatory carcinoma	4.4% (n.a.)
			Axillary dissection in non-invasive carcinoma	12.5% (n.a.)
			Radiotherapy after BCS in invasive carcinoma	4.1% (n.a.)
			Chemotherapy in patients eligible to endocrine therapy	8.7% (n.a.)
Claravezza, 2012 [18]	Italy	2008	Adjuvant chemotherapy in luminal A patients	38% (35–41%)
Poncet, 2009 [19]	France	1999–2003	Trastuzumab in metastatic breast cancer without previous treatment with anthracyclines or in < her-2 negative patients	68% (61–77%)
Lebeau, 2011 [20]	France	2003–2004	Chemotherapy	15% (13–17%)
			Hormonal therapy both in non-metastatic breast cancer, according to appropriateness criteria	9% (7–11%)
Mano, 2010 [21]	Italy	2007	Axillary dissection in DCIS	5% (3–8%)
			Axillary dissection or sentinel lymph node procedure in DCIS or benign lesions	33% (30–37%)
Kiderlen et al., 2015 [22]	Germany, Italy, United Kingdom, The Netherlands, Switzerland, Belgium, Austria	2008–2012	Radical mastectomy in cancer ≤ 3 cm	13% (n.a.)
			ALND in DCIS	4% (n.a.)
Ponti et al., 2007, 2011, 2015 [23–25]	Italy	2011–2012	Axillary staging in cancers other than in pN0	10% (9–11%)
			Axillary dissection in DCIS	3% (2–4%)
			Radical surgery in DCIS < 20 mm	10% (7–12%)
Van de Water, 2012 [26]	The Netherlands	2005–2008	RT in patients aged < 65 years	6% (n.a.)
			RT in patients aged ≥ 75 years	4,5% (n.a.)
			Chemotherapy in patients aged < 65 years	2% (n.a.)
			Endocrine therapy in patients aged < 65 years	2.5% (n.a.)
			Endocrine therapy in patients aged ≥ 75 yearts	7.5% (n.a.)
Lu, 2011 [27]	The Netherlands	1989–2003	Hospital visits in follow-up	31% (28–35%)
			Mammography in follow-up after surgical treatment	18% (16–22%)
Grandjean, 2012 [28]	The Netherlands	2003	Consultations during follow-up	55% (48–62%)
			Mammography during follow-up after surgical treatment	4% (1–7%)
Schwentner, 2013, 2012, 2012 [29–31]	Germany	1992–2008	Surgical management, radiotherapy and chemotherapy in primary breast cancer	Only overall rates of guideline violation are reported, without distinguishing between over and under treatment Guideline violations were 8% for radiotherapy, 13% for surgical management and 16% for chemotherapy
Fong, 2012 [32]	United Kingdom (region of Dundee, Scotland)	2004–2004	Endocrine therapy (comparing observed vs. optimal utilisation rate, predicted from guidelines recommendations)	4% (n.a.)
Ponti, 2014 [33]	Italy, Denmark, Czech Republic, Finland, Ireland, The Netherlands, Norway, Spain, Switzerland (it includes also patients from United States and Japan)	2004–2007	Axillary dissection in DCIS	8% (7–9%)
			Axillary dissection in low/intermediate grade DCIS	5.6% (4.6–7.0%)
			Axillary dissection after breast conserving surgery	4.8% (4–5.5%)
*Colorectal cancer*	*Country*	*Years of care delivery*	*Interventions/procedures*	*Prevalence of overuse* *(95% CI)*
Bonifazi, 2012 [34]	Italy	2006–2007	Bevacizumab use as second-line or advanced line in metastatic colorectal cancer	37% (34–42%)
Adler, 2007 [35]	Germany	Not reported	Diagnostic colonoscopy outside screening programme	14% (11–17%)
Lepage, 2006 [36]	France	2000	Excess of tests executed in pre-operative workup	30% (26–34%)
			Adjuvant chemotherapy in stage III	5% (3–7%)
Eliot, 2014 [37]	Sweden	2000–2010	Pre-operative RT (with or without chemotherapy) in early rectal cancer	55% (50–60%)
*Prostate cancer*	*Country*	*Years of care delivery*	*Interventions/procedures*	*Prevalence of overuse (95% CI)*
Hernes, 2009 [38]	Norway	2004	Radical prostatectomy or Radiotherapy in low risk patients	57% (52–61%)
Grundmark, 2012 [39]	Sweden	1997–2006	Anti-androgen (bicalutamide) in low/intermediate risk patients	2.1% (n.a.)
Evans, 2010 [40]	United Kingdom	Not available	CT scan in diagnostic workup	10% (7–11%)
			Radical prostatectomy	< 1%
			RT	< 1%
			Hormone therapy after diagnosis of prostate cancer	< 1%
*Miscellaneous* ^*a*^	*Country*	*Years of care delivery*	*Interventions/procedures*	*Prevalence of overuse*
Ray-Coquard, 2012 [41]	France	2010	Erythropoiesis stimulating agents in chemotherapy-induced anaemia	Overall prevalence of overuse 5% in breast (*n* = 185) and lung (*n* = 227) cancer patients (20/412)
Joerger, 2014 [42]	Switzerland	2012	Anticancer drugs in several cancers, including breast, lung and colorectal	Results by cancer site reported only in graphic Overall, 32% of all patients received at least one off-label drug, but off-label use was unsupported by European Society for Medical Oncology guidelines only in 6.6% of cases Inappropriate use was higher for bevacizumab (29%) also because of its use in advanced breast cancer

^a^Studies including patients with different cancers are in this category

*ALND* axillary lymph nodes dissection, *BCS* breast conserving surgery, *DCIS* ductal carcinoma in situ, n.a. not available, *RT* radiotherapy

**Table 3:** Prevalence of overuse in European studies on patterns of care for breast, colorectal, lung and prostate cancer, according to type of procedure^a^

Type of procedure	Prevalence (median)	Range	Number of measures
Drugs	15%	2–68%	14 from 12 studies
Diagnostic	24%	4–55%	8 from 5 studies
Surgery	8%	3–57%	13 from 7 studies
Radiotherapy	5%	4–24%	5 from 4 studies

^a^Two studies [29–31, 42] not included as prevalence of overuse was neither explicitly reported, nor extractible from tables

**Table 4:** Prevalence of overuse in European studies on patterns of care for breast cancer, according to type of procedure^a^

Type of procedure	Prevalence (median)	Range	Number of measures
Drugs	8%	2–68%	8 from 6 studies
Diagnostic	25%	4–55%	4 from 2 studies
Surgery	8%	3–34%	11 from 5 studies
Radiotherapy	4.5%	4–7%	3 from 2 studies

^a^One study [29–31] not included as prevalence of overuse was neither explicitly reported, nor extractible from tables

Orientation towards overuse did not increase over time, with overuse being directly measured in 24% of the studies published before 2010, and only in 13% of those published in 2015–2016 (Table 5). Not surprisingly, individual studies’ conclusions were related to the approach employed in assessing processes of care. Overall, in 26 studies, the authors explicitly stated the identification of some degree of overuse for the procedures/interventions investigated; in 19 of those cases, the conclusions were drawn from overuse measures, whereas in the remaining 7 cases they relied on variation analysis of generic utilisation rates (Table 5).

**Table 5:** Measures adopted in European studies on patterns of care for breast, colorectal, lung, and prostate cancer, according to year of publication and to the conclusions drawn by their Authors

Measures		Year of publication					Authors’ conclusions				
		≤ 2010 N (%)	2011–2012 N (%)	2013–2014 N (%)	2015–2016 N (%)	Total N (%)	No explicit statement N (%)	Underuse N (%)	Overuse N (%)	Under and overuse N (%)	Total N (%)
Generic	N %	16 (55.2) (33.3)	8 (32) (16.7)	12 (48) (25)	12 (80) (25)	48 (51) (100)	24 (69) (50)	17 (51.5) (35.4)	5 (41.7) (10.4)	2 (14.3) (4.2)	48 (51) (100)
Oriented towards underuse	N %	6 (20.7) (25)	8 (32) (33.3)	9 (36) (37.5)	1 (7) (6.7)	24 (25.5) (100)	8 (23) (33)	16 (48.5) (67)	0 (0) (0)	0 (0) (0)	24 (25.5) (100)
Oriented towards overuse	N %	7 (24.1) (31.8)	9 (36) (40.9)	4 (16) (18.2)	2 (13) (9.1)	22 (23.5) (100)	3 (8) (13.6)	0 (0) (0)	7 (58.3) (31.8)	12 (85.7) (54.5)	22 (23.5) (100)
Total	N %	29 (100) (31.2)	25 (100) (25.8)	25 (100) (26.9)	15 (100) (16.1)	94 (100) (100)	35 (100) (36.6)	33 (100) (35.5)	12 (100) (12.9)	14 (100) (15)	94 (100) (100)

## Discussion

The value of research on quality of care should be judged on the extent to which it provides useful information to guide the decisions and actions of those responsible for monitoring and improving healthcare delivery. Any substantial mismatch between the information supplied by researchers and the one actually demanded by ‘research users’ implies failure in meeting this goal.

The issue of overuse in the delivery of healthcare has been steadily emerging over the last 10 years as particularly relevant due to its many implications for quality and safety, and for the economic sustainability of healthcare systems. There is indeed a growing expectation that withdrawing resources from the delivery of care with no or little clinical value, and reducing the inappropriate utilisation of otherwise clinically valuable interventions, will improve effectiveness and efficiency [1–3, 43–51]. Overuse of diagnostic and therapeutic procedures/interventions has also been identified as an issue in cancer care [6, 52]. Therefore, from this perspective, a key criterion to assess the actual informative value of research on quality of care is its ability to identify areas of overuse and provide estimates of its prevalence.

Indeed, on these grounds, research in the area of cancer care is at risk of being of little informative value. According to our findings, overuse has been addressed only in a few studies, and on a limited number of procedures and interventions. We found that only 22 (approximately a quarter of the total number of studies identified) were concerned with overuse. Even more importantly, our results highlight that the attention towards overuse has not changed over time, and has certainly not increased as would have been expected given its growing policy concerns.

Our findings are in line with what has been documented in previous reviews. Further, despite its policy relevance, information on the extent to which overuse actually permeates clinical practice has been shown to be relatively scant and unsystematic, having been addressed only by relatively few studies and on a limited number of procedures and interventions across different practice areas [16, 53, 54]. Understanding the reasons behind such limited attention towards the problem of ‘too much care’ seems to be important to the design of future research initiatives better equipped to address the challenges and implications of overuse facing health policy.

Although the limited literature available on overuse has been justified mainly on technical grounds, namely the lack of reliable and scientifically sound measures of overuse [55], as others have pointed out, there may also be cultural and political barriers to the “*willingness*” to address overuse [56]. Nevertheless, the relevance of these technical aspects cannot be dismissed. Identification of overuse in clinical practice can be hampered by a number of factors, including the lack of explicit standards (i.e. from practice guideline recommendations or appropriateness criteria) against which actual care should be compared, and the lack of sufficiently detailed information on patient characteristics; the latter is required in order to identify the specific clinical indications in which an intervention/procedure has been used.

The insufficient and unsatisfactory development of measures properly aimed at detecting the delivery of low-value care has been previously discussed [55], as has the need to address overuse in the process of guideline development through explicitly negative recommendations [57]. According to our best knowledge, only the National Institute for Health and Care Excellence (NICE) in the United Kingdom has systematically taken such an approach, developing negative recommendations stating which interventions should not be offered in specific clinical indications [58, 59]. The internationally known ‘Choosing Wisely’ campaign [60] offers lists of low-value interventions and of procedures at risk of being used inappropriately, but those lists would need to be translated into measures or process indicators with explicit denominators and numerators [54].

Nevertheless, while the lack of measures and the issues faced when gathering the necessary information may play a role in constraining the empirical assessment of overuse, they surely do not seem to represent the only possible explanation of such limited attention being devoted to this issue.

Explicit standards were employed in 46 studies, approximately half of our sample, and although only 22 of them used these standards to measure overuse, the remaining studies could have done the same if a different sampling approach had been adopted. Indeed, the studies using direct measures of overuse did not differ from others in terms of the sources of data on which they relied. Only 13 studies relied on data drawn from administrative databases, thus being potentially limited in their ability to gather detailed information on individual patient characteristics, while all others had the opportunity to rely on data drawn from multiple and often integrated sources.

Further, aside from the abovementioned technical reasons, the neglect to address the issue of overuse might be explained on other grounds [56] related to the dominant cultural attitude of health professionals dealing with cancer care, much more sympathetic to the problem of cancer patients not having access to the treatment they need rather than to the one represented by exposing patients to ‘too much care’. Indeed, an indirect sign of the propensity to assign more importance to underuse compared to overuse is that, when generic rates were adopted to analyse variations in patterns of care, authors more frequently referred only to the issue of some patients not receiving appropriate care, rather than the concurrent problem of patients being exposed to ‘too much care’ (Table 5), despite the procedures analysed being recommended for specific clinical indications. Finally, the alternative hypothesis is that health services overuse in cancer care in Europe does not actually represent a priority, with other dimensions of quality in healthcare delivery being more relevant. Indeed, a recent report on improving efficiency in cancer care left the problem of unnecessary use of healthcare interventions largely unaddressed [61].

Our study has its limitations. Our search was not systematic, as we did not aim to identify all the published studies on quality of care for cancer patients. Rather, we aimed to conduct a systematic review to identify a representative sample of studies published over the last 10 years to assess whether the literature allowed the identification of areas of cancer care more likely to be exposed to the problem of overuse. The methodological approach adopted is close to a scoping review [62, 63], an approach increasingly used when the aim is to provide an overall description and analysis of the available literature in a field or on a specific topic, thus providing a map of the basic features of the studies conducted in that research area. Therefore, the literature search we undertook is far from being fully comprehensive (we limited our search to Medline), and it is likely that we missed potentially relevant papers. However, our findings should be primarily judged in the light of the representativeness of the study sample analysed. From this perspective, we do not believe that a more extensive literature search would have made our findings substantially different, as it is unlikely that studies retrievable from other databases or available in the grey literature would be systematically different from those identified.

Further, our study selection criteria can, of course, be questioned. We did not include studies aimed at assessing the impact of quality improvement interventions, as their primary goal was not of describing patterns of care. Nevertheless, one may argue that we missed potentially relevant information on over- or under-utilisation, at least by not considering the baseline (i.e. pre-intervention) rates provided in those studies. However, we deemed baseline rates of questionable generalisability (being observed in individual centres ‘selected’ as the target of the quality improvement efforts), and that, overall, extrapolation of data on over/underuse would have required a high degree of subjective interpretation. Nevertheless, had we included those studies, as quality improvement efforts have been thus far rarely aimed at de-implementing procedures/interventions, it is likely that the proportion of overuse-oriented studies would have been even lower than the observed.

Overall, despite the limitations of our search, it is reasonable to consider the studies included in our review as a sufficiently representative sample of the European studies on the quality of cancer care conducted over the time-frame considered.

## Conclusion

Among the European studies aimed at describing the processes of care delivered to cancer patients and published between 2006 and 2016, few were explicitly oriented to measuring overuse. This finding is at odds with the proliferation of initiatives aimed at tackling the issue of low-value care we have been witnessing over the last 10 years, both from government and research and professional bodies. Such increasing attention to the problem of overuse in the policy context does not seem to be mirrored by a similar attitude in studies on quality of care for cancer patients, presumably due to weak connections between researchers in this area and policy circles.

From this perspective, the overall picture emerging from our description of the literature in this field highlights another example of mismatch between research and policy. While policy-makers are concerned with the sustainability of health systems, through the reduction of clinical waste among other measures, research provides only scant information to guide their efforts. Policy aimed at withdrawing resources from inappropriately used interventions to better support the delivery of high-value care would need reliable information on prevalence and determinants of overuse in order to set priorities on which it could be worthwhile to focus the development of targeted policy initiatives. According to our findings, current research in the area of assessment and evaluation of quality of cancer care is probably of little help in providing such guidance, and the call for overuse being a major topic of research initiatives [57] is indeed supported by our findings. This mismatch between research and policy does not have an easy solution, and calls for institutional actions aimed at improving the connections between research and policy [64] and for the development of initiatives explicitly aimed at fostering debate and discussion among researcher and policy-maker communities [14, 65].

## Additional files

Additional file 1: Search strategy adopted for the identification of studies on patterns of care for breast, colorectal, lung and prostate cancer, published between 2006 and 2016. (DOCX 23 kb)

Additional file 2: European studies on patterns of care for breast, colorectal, lung and prostate cancer included in the review. (DOCX 246 kb)
