# Supplementary material for: Overuse in cancer care: do European studies provide information useful to support policies?
Source: Health Res Policy Syst. 2018 Feb 20;16:12. doi: 10.1186/s12961-018-0287-z (PMC5819192; doi:10.1186/s12961-018-0287-z)
Supplement: Supplementary file 1 — Search strategy adopted for the identification of studies on patterns of care for breast, colorectal, lung and prostate cancer, published between 2006 and 2016. (DOCX 23 kb) [file 12961_2018_287_MOESM1_ESM.docx]

**Additional file 1**

**Search strategy adopted for the identification of studies on patterns of care for breast, colorectal, lung, and prostate cancer, published between 2006-2016**

**SEARCH STRATEGY**

**Entire query:**

Search (((((((((cancer[MeSH Major Topic]) OR tumor[MeSH Major Topic]) OR neoplasm[MeSH Major Topic]) AND ( "2006/06/14"[PDat] : "2016/06/14"[PDat] ))) AND (((((((lung[tiab]) OR colon[tiab]) OR colorectal[tiab]) OR rect*[tiab]) OR breast[tiab]) OR prostate[tiab])) AND ((((((((((((((((((((((((((((((disinvest*[tiab]) OR low-value[tiab]) OR "choosing wisely"[tiab]) OR overuse[tiab]) OR over-use[tiab]) OR appropriateness[tiab]) OR inappropriateness[tiab]) OR underuse[tiab]) OR under-use[tiab]) OR "over utilization"[tiab]) OR "over-utilization"[tiab]) OR overutilization[tiab]) OR "over utilisation"[tiab]) OR "over-utilisation"[tiab]) OR "overutilisation"[tiab]) OR "under utilization"[tiab]) OR "under-utilization"[tiab]) OR underutilization[tiab]) OR "under utilisation"[tiab]) OR "under-utilisation"[tiab]) OR underutilisation[tiab]) OR under-prescription[tiab]) OR underprescription[tiab]) OR over-prescription[tiab]) OR overprescription[tiab]) OR Overtreat*[tiab]) OR Undertreat*[tiab]) AND ( "2006/06/14"[PDat] : "2016/06/14"[PDat] ))) OR (((((((Quality assurance[MeSH Major Topic]) OR Quality control[MeSH Major Topic]) OR Continuous quality improvement[MeSH Major Topic]) OR Guideline adherence[MeSH Major Topic]) OR Healthcare quality indicators[MeSH Major Topic]) OR Total quality management[MeSH Major Topic]) AND ( "2006/06/14"[PDat] : "2016/06/14"[PDat] ))) AND ( "2006/06/14"[PDat] : "2016/06/14"[PDat] ))) NOT (((((((("case reports"[Publication Type]) OR "clinical trial"[Publication Type]) OR "comment"[Publication Type]) OR "editorial"[Publication Type]) OR "review"[Publication Type]) OR "letter"[Publication Type]) OR "meta analysis"[Publication Type]) OR "randomized controlled trial"[Publication Type]))) AND "english"[Language]

**Search component:**

1. Appropriateness

2. Quality of care

3. Cancer

4. Most frequent cancers

5. Study design

Components 1, 2, 3, 4 were combined with “*AND*”, while component 5 has been combined in the strategy with “*NOT*”. The strategy was limited from 14^th^ June 2006 to 14^th^ June 2016 and with English language “*eng[Language]*”.

**Records found:**

Records identified through PUBMED searching: n = **1857** (to 29^th^ June 2016)

**Search strategy Builder:**

| **Search component 1: APPROPRIATENESS** |  | **Search component 2: QUALITY OF CARE** |  | **Search component 3: CANCER** |  | **Search component 4: MOST FREQUENT CANCERS** |  |  | **Search component 6: STUDY DESIGN** |
| --- | --- | --- | --- | --- | --- | --- | --- | --- | --- |
|  | ***AND*** |  | ***AND*** |  | ***AND*** |  |  | ***NOT*** |  |
| disinvest*[tiab] |  | Quality assurance[MeSH Major Topic] |  | cancer[MeSH Major Topic] |  | lung[tiab] |  |  | "case reports"[Publication Type]) |
| low-value[tiab] |  | Quality control[MeSH Major Topic] |  | neoplasm[MeSH Major Topic] |  | colon[tiab] |  |  | "clinical trial"[Publication Type] |
| "choosing wisely"[tiab] |  | Continuous quality improvement[MeSH Major Topic] |  | tumor[MeSH Major Topic] |  | colorectal[tiab] |  |  | "comment"[Publication Type] |
| overuse[tiab] |  | Guideline adherence[MeSH Major Topic] |  | ***combined with “OR”*** |  | rect*[tiab] |  |  | "editorial"[Publication Type] |
| over-use[tiab] |  | Healthcare quality indicators[MeSH Major Topic] |  |  |  | breast[tiab] |  |  | "review"[Publication Type] |
| appropriateness[tiab] |  | Total quality management[MeSH Major Topic] |  |  |  | prostate[tiab] |  |  | "letter"[Publication Type] |
| inappropriateness[tiab] |  | ***combined with “OR”*** |  |  |  | ***combined with “OR”*** |  |  | "meta analysis"[Publication Type] |
| underuse[tiab] |  |  |  |  |  |  |  |  | "randomized controlled trial"[Publication Type] |
| under-use[tiab] |  |  |  |  |  |  |  |  | ***combined with “OR”*** |
| "over utilization"[tiab] |  |  |  |  |  |  |  |  |  |
| "over-utilization"[tiab] |  |  |  |  |  |  |  |  |  |
| overutilization[tiab] |  |  |  |  |  |  |  |  |  |
| "over utilisation"[tiab] |  |  |  |  |  |  |  |  |  |
| "over-utilisation"[tiab] |  |  |  |  |  |  |  |  |  |
| "overutilisation"[tiab] |  |  |  |  |  |  |  |  |  |
| "under utilization"[tiab] |  |  |  |  |  |  |  |  |  |
| "under-utilization"[tiab] |  |  |  |  |  |  |  |  |  |
| underutilization[tiab] |  |  |  |  |  |  |  |  |  |
| "under utilisation"[tiab] |  |  |  |  |  |  |  |  |  |
| "under-utilisation"[tiab] |  |  |  |  |  |  |  |  |  |
| underutilisation[tiab] |  |  |  |  |  |  |  |  |  |
| overprescription[tiab] |  |  |  |  |  |  |  |  |  |
| over-prescription[tiab] |  |  |  |  |  |  |  |  |  |
| underprescription[tiab] |  |  |  |  |  |  |  |  |  |
| under-prescription[tiab] |  |  |  |  |  |  |  |  |  |
| Overtreat*[tiab] |  |  |  |  |  |  |  |  |  |
| Undertreat*[tiab] |  |  |  |  |  |  |  |  |  |
| ***combined with “OR”*** |  |  |  |  |  |  |  |  |  |
