# Supplementary material for: Overuse in cancer care: do European studies provide information useful to support policies?
Source: Health Res Policy Syst. 2018 Feb 20;16:12. doi: 10.1186/s12961-018-0287-z (PMC5819192; doi:10.1186/s12961-018-0287-z)
Supplement: Supplementary file 2 — European studies on patterns of care for breast, colorectal, lung and prostate cancer included in the review. (DOCX 246 kb) [file 12961_2018_287_MOESM2_ESM.docx]

**Additional file 2**

**European studies on patterns of care for breast, colorectal, lung, and prostate cancer, included in the review**

**Additional file 2a: Breast cancer**

|  | **Country** | **Years of care delivery, sample size, study design, source of information** | **Explicit standard** | **Measures** | **Interventions/procedures** | **Prevalence of overuse (95%CI)** | **Notes** |
| --- | --- | --- | --- | --- | --- | --- | --- |
| Fedeli, U., et al., (2007) (1) | Italy | 2000 – 2004  18584  Cross-sectional  Administrative database | No | Generic rates | BCS |  | Overall rate of BCS was 61.5%, ranging from 56% in 2000, to 67% in 2004. Rates were lower in the elderly. |
| Ponti, A., et al., (2007, 2011, 2015) (2-4) | Italy | 2011 – 2012  7284  Cross sectional  Specialized database | yes | Rates of not eligible exposed to the procedures (C/C+D) | Axillary staging in cancers other than in pN0  axillary dissection in DCIS  radical surgery in DCIS < 20 mm | 10% (9% - 11%)  3% (2% - 4%)  10% (7% - 12%) |  |
| Brucker, S. Y., et al., (2008, 2011) (5, 6) | Germany | 2003 – 2009  31656 (in 2007)  Cross-sectional  Specialized database | Yes | Rates of eligible treated according to guidelines(a / a+b) | BCS, post surgery RT, first line chemo and endocrine therapy, axillary node dissection, SLNB |  | In the last year of data collection rates were 90% for appropriate* axillary dissection, 74% for SLNB, 97% for endocrine therapy , 72% for use of standard regimens in chemotherapy, 75% for adjuvant and neo-adjuvant chemotherapy, 79% and 85% and 70% for radiotherapy after breast conserving surgery and after mastectomy, respectively. Breast conserving surgery in ranged from 13% to 85%, according to tumour stage (mean 70%) |
| Poncet, B., et al., (2009) (7) | France | 1999-2003  131  cohort retrospective  clinical charts | Yes | Rate of procedures performed according to guidelines (a/a+c) | Trastuzumab | 79% (71% - 86%) |  |
| Rosato, R., et al., (2009) (8) | Italy | 2000-2004  16022  cohort retrospective  administrative database | No | Generic rates | BCS with or without RT  Mastectomy | Not available | 20,3% pts received BCS without RT. Underuse of RT after BCS, found to be related to patient (age, presence of comorbidities, living alone) and hospital characteristics ( distance from place of residence, level of specialisation). |
| Allemani, C., et al., (2010) (9) | Danmark, The Netherlands, Spain, Italy, Finland, Poland, Estonia, France, Slovakia, Slovenia, Sweden, Iceland | 1996-1998  13485  cross sectional  cancer registries + clinical records | No | Rates of eligible treated according standard care (a / a+b) | Rate of BCS + RT, Adjuvant chemo in N+ 15-49 year, Adjuvant chemo in N+ 50-99 years, Endocrine therapy in ER+ 15-49 years, Endocrine therapy in ER+ 50-99 years old. |  | Rates were 55% for BCS+ RT ; 90.7% for adjuvant chemo in N+ 15-45 year old ; 52.1% adjuvant chemo in N+ 50-99 year old ; 44.6% for endocrine therapy in ER+ 15-49 years ; 58.8 % for endocrine therapy in ER+ 50-99 years . Striking variation across countries |
| Ess, S., et al., (2010) (10) | Switzerland | 2003 – 2005  4820  cross-sectional  cancer registry+clinical records | Yes | Generic rates | BCS, mastectomy, sentinel procedure, RT after BCS and after mastectomy, Endocrine therapy, chemotherapy in endocrine unrensponsive disease | Not available | Rates were  66% for BCS, 30% for mastectomy (4% with breast reconstruction), 46% sentinel procedure, 92% RT after BCS, 66% RT after mastectomy, 91% endocrine therapy and 83% chemotherapy in endocrine unresponsive disease. Variations observed mostly in use of RT after mastectomy and to a lower but still statistically significant extent for endocrine therapy. |
| Jeevan, R., et al., (2010) (11) | UK | 2006 – 2009  44837  cross-sectional  administrative database | Yes | Generic rate | Breast reconstruction after mastectomy | Not available | Rate of breast reconstruction was 16.5% over the study period. Substantial regional variation not explained by the characteristics of the local patient population (age, comorbidity, deprivation, ethnicity) . |
| Mano, M. P., et al., (2010) (12) | Italy | 2000-2007  3432  cross sectional  clinical charts | Yes | Rates of not eligible exposed to the procedures (C/C+D) | Axillary dissection in Ductal Carcinoma in situ (DCIS)  Axillary dissection or sentinel lymph node procedure in DCIS or benign lesions | 5% (3% - 8%)  33% (30% - 37%) |  |
| Montemurro, F., et al., (2010) (13) | Italy | 1999-2008  321  cross sectional  ah hoc data collection | No | Rates of eligible treated according to guidelines (a / a+b) | Use of anthracycline in patients receiving trastuzumab-based therapy |  | Overall rate of anthracycline use was 21% |
| van Steenbergen, L. N., et al., (2010) (14) | The Netherlands | 2003-2006  From 1240 in 2003 to 13280 in 2006  Cohort retrospective  Cancer registry | Yes | Rates of eligible treated according to guidelines (a / a+b) | BCS, RT after BCS, SLNP, axillary lymph nodes dissection, adjuvant chemo or hormone therapy |  | BCS in T1T2 N0 ranged from 30% to 82% , almost always followed by RT. Overall rate of use of SLNP was 22% in 2003 and 78% in 2006 in N0 patients, paralleled by a decrease in axillary lymph nodes dissection (from 22% to 11%). Increase over time in the rate of use of adjuvant treatments. Overall, wide variation across hospitals. |
| Wockel, A., et al., (2010) (15) | Germany | 2001 – 2005  3976  cohort retrospective  not clear | Yes | Rate of procedures performed according to guidelines (a/a+c) | BCS  axillary dissection  radiotherapy  chemotherapy | 4.4% - ( ?)  12.5% -( ?)  4.1% - ( ?)  8.7% ( ?) |  |
| Bastiaannet E., et al., (2011) (16) | The Netherlands | 1995 – 2005  127805  Retrospective cohort  Cancer registry | No | Generic rates | Surgery, adjuvant chemotherapy, hormonotherapy, RT after breast conserving surgery |  | Patterns of care are analysed over time in different patients’age. A potential overuse of hormonotherapy in the elderly, along with underuse of surgical treatment and adjuvant chemotherapy is highlighted |
| Lebeau, M., et al., (17)(2011) | France | 2003-2004  926  cross-sectional  clinical charts | Yes | Rate of procedures performed according to guidelines (a/a+c) | Chemotherapy  Hormonal therapy | 15% (13% - 17%)  9% (7% - 11%) |  |
| Lu, W., et al., (2011)(18) | The Netherlands | 1989-2003  662  cohort retrospective  cancer registry and clinical charts | yes | Rates of not eligible exposed to the procedures (C/C+D) | Hospital visits in follow-up  Mammography in follow-up | 31% (28% - 35%)  18% (16% - 22%) |  |
| Struikmans, H., et al., (2011) (19) | The Netherlands | 1997 – 2008  65966  cohort retrospective  cancer registry + administrative database | No | Generic rates | BCS, mastectomy, RT after BCS |  | Proportion of pts receiving BCS ranged from 32-45% in 1997 and from 41-57% in 2008 Frequencies of RT after BCS were between 90-99% in 1997 and 96-98% in 2008. |
| Weggelaar, I., et al., (2011) (20) | The Netherlands | 2001 – 2006  2336  cross sectional  cancer registry | Yes | Rates of eligible treated according standard care (a / a+b) | Rate of guidelines adherence as for surgical treatment, RT, Systemic therapy, Chemotherapy, Endocrine therapy |  | Overall, rates of compliance with guidelines was 91% for surgical treatment, 91% for RT, 77% for systemic therapy, 90% for chemotherapy, and 79.7% for endocrine therapy. Rates were substantially lower in older patients. |
| Clavarezza, M., et al., (2012) (21) | Italy | 2008  1894  cross-sectional  ad hoc data collection | Yes | Rate of procedures performed according to guidelines (a/a+c) | adjuvant chemotherapy in Luminal A patients | 38% (35% – 41%) |  |
| Fong, A., et al., (2012) (22) | UK (region of Dundee, Scotland) | 2000 – 2004  1743  Cohort retrospective  Cancer registry | Yes (estimates of “optimal utilisation rates” based upon epidemiological data and guidelines’recommendations) | Rate of procedures performed according to guidelines (a/a+c) | Endocrine therapy | 4% (? - ?) |  |
| Grandjean, I., et al., (2012) (23) | The Netherlands | 2003  198  Cohort retrospective  Cancer registry + clinical records | yes | Rates of eligible treated(a / a+c) | Clinical consultations during follow-up  Mammography during follow-up | 55% (48% - 62%)  4% (1% - 7%) |  |
| Schwentner L., et al., (2012, 2013) (24-26) | Germany | 1992- 2008  9156  Cohort retrospective  Specialized database | Yes | Rate of procedures performed according to guidelines (a/a+c) | Surgical management,  Radiotherapy,  chemotherapy |  | Only overall rates of guideline violation are reported, without distinguishing between over and under treatment. Guideline violations were 8% for radiotherapy, 13% for surgical management, and 16% for chemotherapy |
| Stordeur, S., et al., (2012) (27) | Belgium | 2001 – 2006  50,039  Cross-sectional  Cancer registry + administrative database | Yes | Rates of eligible treated(a / a+b) | Two-view mammography or breast ultrasonography within 3 months before surgery in newly diagnosed stage I-III; ER and PgR status assessment before systemic treatment ; cytological or histological assessment before surgery; BCS, adjuvant chemotherapy within 4 months after surgery, adjuvant hormone therapy within 9 months after surgery, Rt after BCS, systemic chemotherapy as first or second line treatment in metastatic cancer; number of mammograms per patients 1 year after last treatment |  | Rates were as follows in 2006:  Two.view mammography or breast ultrasonography within 3 months before surgery in newly diagnosed stage I-III: 86%; ER and PgR status assessment before systemic treatment 98%; cytological or histological assessment before surgery: 71%;  BCS 58%; adjuvant chemotherapy 37%; adjuvant hormone therapy 43%; Rt after BCS 90%; , systemic chemotherapy as first or second line treatment in metastatic cancer 84%; number of mammograms per patients 1 year after last treatment (mean) :1.73 |
| van de Water, W., et al., (2012) (28) | The Netherlands | 2005 – 2008  24959  cohort retrospective  cancer registry + administrative database | Yes | Rate of procedures performed according to guidelines (a/a+c) | RT in < 65 year patients  RT in =>75 year patients  Chemotherapy in <65 year patient  Endocrine therapy in <65 year patient  Endocrine therapy in =>75 year patients | 6% (? - ?)  4,5% (? - ?)  2% (? - ?)  2,5% (? - ?)  7,5% (? - ?) |  |
| Vrijens F., et al., (2012) (29) | Belgium | 2004 – 2006  25178  Cohort retrospective  Cancer registry + administrative database | Yes | Rates of eligible treated according to guidelines (a / a+b) | BCS, neoadjuvant treatment, adjuvant chemotherapy, adjuvant hormone therapy, Rt after BCS, systemic chemotherapy as first or second line treatment in metastatic cancer; mammography after history of breast cancer |  | Rates were as follows: BCS 69%; neoadjuvant treatment in operable cT2-T3 15%; adjuvant chemotherapy 37%; adjuvant hormone therapy 43%; Rt after BCS 89%; , systemic chemotherapy as first or second line treatment in metastatic cancer 88%; mammography after history of breast cancer 81%. |
| Joerger, M., et al., (2013) (30) | Switzerland | 2003 – 2005  4820  Cohort retrospective  Cancer registry + clinical records | Yes | Rates of not eligible exposed to the procedures (C/C+D) | Mastectomy | 15% (13% - 16%) |  |
| Leoni, M., et al., (2013) (31) | Italy | 2002-2005  10024  Cohort retrospective  Administrative data | No | Generic rates | Use of Mammography, Abdominal echogram, Bone scan, and Chest x-ray, in follow-up after surgery |  | Overall rates within 18 months after surgery were 72.1% for mammography, 64% for abdominal echogram, 38% for bone scan, and 52% for chest x-ray. The last there rates were substantially higher than in the general female population. The observed variation among units supports the overtesting hypothesis. |
| Arican, A., et al., (2014) (32) | Turkey | 2010-2011  1038  cross-sectional  not clear | Yes | Rates of eligible treated according to guidelines (a / a+b) | Diagnosis of bone metastasis (radiology, interventional diagnosis, laboratory tests)  Staging (laboratory tests and radiology)  Treatment (surgery, radiotherapy, chemo or hormonal therapy) |  | Compliance with guidelines was  Diagnosis : 95% for radiology, 3.5% for interventional diagnosis, 98% per laboratory tests)  Staging : 99% laboratory tests; 96% radiology  Treatment : 31% surgery; 56% radiotherapy ; 97% chemotherapy and hormonal therapy |
| Gion, M., et al., (2014, 2016) (33, 34) | Italy | 2012  Not Applicable  Cross-sectional  Administrative database | No | Generic rates | Use of marker Ca15.3 |  | Observed rates of us of Ca12.5 were compared to expected one based on incidence and prevalence data and guidelines recommendations. Contrary to what observed for other markers considered in the study and related to the management of other tumours, the amount of potential overuse observed for Ca12.5 in breast cancer was limited. |
| Ponti, A., et al., (2014) (35) | Czech Republic, Denmark, Finland, Italy, Ireland, Norway, Spain, Switzerland (and USA) | 2004 - 2008  5324  Cross-sectional  Specialized database | Yes | Rates of not eligible exposed to the procedures (C/C+D) | Axillary dissection in DCIS  Axillary dissection in low/intermediate grade DCIS  Axillary dissection after breast conserving surgery | 8% (7% - 9%)  5.6% (4.6% - 7.0%)  4.8% (4% - 5.5%) |  |
| Dialla, P. O., et al., (2015) (36) | France | 1998 – 2009  4305  Prospective cohort  Cancer registry | No | Generic rates | BCS  Mastectomy  Chemotherapy  Hormone therapy |  | Relatively less frequent use of BCS and adjuvant therapy in older patients. Undertreatment associated with worse survival rates |
| Invald EC 2014(37) | Germany | 2000-2012  6164  Cohort retrospective  Cancer registry | Yes | Rates of eligible treated according to guidelines (a / a+b) | Chemotherapy (trastuzumab)  Hormone therapy |  | Overall, in HER-2 positive patients, Trastuzumab was used in 43.6% and 30.9% of those in premenopausal and postmenopausal status, respectively. In premenopausal women, rate of trastuzumab use increased over time, from 58% in 2006, to 91% in 2011. In postmenopausal women, proportion of those receiving trastuzumab remained stable (50%) .  Hormone therapy used in 85% of those Steroid Hormone Receptor-positive.  A considerable number of HER-2 positive patients found not receiving adequate treatment. |
| Kelly, E., et al., (2015) (38) | Denmark, England, Finland, France, Iceland, The Netherlands, Norway, Sweden (and Australia) | 2001-2002  NA  cross-sectional  administrative data | No | Generic rates | Endocrine therapy |  | Cross-country comparison of prescription rates, showing use of endocrine therapy remaining stable or increasing over time, with France and England having the highest rates, and Norway the lowest, also taking into account differences in breast cancer incidence. Cross-country variation in type of endocrine therapy used were also observed. |
| Kiderlen, M., et al., (2015) (39) | Germany, Italy, UK, The Netherlands, Switzerland, Belgium, Austria | 2003-2012  41.871  Cross-sectional  Specialized database | Yes | Rates of not eligible exposed to the procedures (C/C+D) | Radical mastectomy in cancer <3 cm  ALND in DCIS | 13% ( ? - ?)  4% (? - ?) |  |
| Massat, N. J., et al., (2015) (40) | UK | 2011-2012  Not Applicable  Cross-sectional  Administrative databases | No | Generic rates | Screening mammography in women (50-64 years) |  | Overall rate was 75.6% with significant variability primarily explained by populations factors |
| Morgan, J., et al., (2015) (41) | UK | 2002-2010  1729  Cohort retrospective  Cancer registry+ administrative data | No | Generic rates | Surgery in patients > 70 with ER+ operable cancer |  | Overall rate was 58%, with variations across hospitals suggesting that some women may be undertreated or overtreated, |
| Gondos, A., et al., (2016) (42) | Denmark  The Netherlands  Sweden  Belgium  Norway  Germany  Italy | 2003-2010  14109  Cross-sectional  Cancer registries | No | Generic rates | Sentinel lymph node technique  Axillary dissection |  | In 2003, sentinel lymph node biopsy (SLNB) use varied between 26% and 81% for pT1 tumors, and between 2% and 68% for pT2 tumors. By 2010, SLNB use increased to 79-96% and 49-92% for pT1 and pT2 tumors, respectively. Axillary lymph node dissection (ALND) use for pT1 tumors decreased from between 75% and 27% in 2003 to 47% and 12% in 2010, and from between 90% and 55% to 79% and 19% for pT2 tumors, respectively. Persisting variation in important aspects of axillary management |
| Richards, P., et al., (2016) (43) | UK | 2002- 2010  17129  Cohort retrospective  Cancer registry+ administrative database | Yes | Rates of eligible treated according to guidelines (a / a+b) | Rate of surgery in ER+ early stage patients >70 |  | Overall rate of surgery was 58% over the whole study period. Surgery underused, with no evidence of change over time. Age was an important predictor of whether or not a woman received surgery, as well as co-morbidity, deprivation, symptomatic presentation, later stage at diagnosis and low grade |
| Rococo, E., et al., (2016) (44) | France | 2012  63414  Cross-sectional  Administrative database | No | Generic rates | BCS  Breast reconstruction after mastectomy  Sentinel lymph node biopsy |  | Rates were 74% (for BCS, 8% for immediate breast reconstruction; 39% for sentinel lymph node biopsy. Considerable variation in immediate breast reconstruction and SLNB rates among hospitals |

**Additional file 2b: Colorectal cancer**

|  | **Country** | **Tumor, years of study conduct, sample size, study design, source of information** | **Explicit standard** | **Measures** | **Interventions/procedures** | **Prevalence of overuse (95%CI)** | **Notes** |
| --- | --- | --- | --- | --- | --- | --- | --- |
| Lepage, C., et al., (2006)(45) | France | Colon  2000  567  cross-sectional  cancer registry and hoc-data collection | Yes | Rate of procedures performed according to guidelines (a/a+c) | Excess of tests executed in pre-operative workup  Adjuvant chemotherapy in stage III | 30% (26% - 34%)  5% (3% - 7%) |  |
| Adler, A., et al., (2007) (46) | Germany | Colorectal  ?  1397  cross-sectional  ad hoc data collection | Yes | Rate of procedures performed according to guidelines (a/a+c) | Diagnostic colonscopy | 14% (11% - 17%) | 86% of colonscopy were appropriate according to ASGE criteria, and 63% according to EPAGE criteria. |
| Morris, E., P. et al., (2008) (47) | UK | Rectal  1998-2004  31223  cross-sectional  cancer registry + administrative data | No | Generic rate | Rates of abdominoperineal excision |  | Over the study period the use of surgical techniques less demolitive than APE increased, with APE rates decreasing from 30.5% to 23% . Nevertheless, statistically significant variation was observed in APE rates.. |
| Kube, R., et al., (2009) (48) | Germany | Colorectal  2000-2004  5936 (in 2004)  Cohort retrospective  Ad hoc data collection | No | Generic rates | Curative resection; abdominoperineal resection in rectal cancer; neoadjuvant radio e radiochemotherapy in rectal cancer; anastomotic leakage; mesorectal excision; |  | Rates of curative resection in 2004 were 82% and 86%, for colon and rectal cancer, respectively. Rate of abdominoperineal resection in rectal was 22%. Neoadjuvant radio or radiochemotherapy was used in 25% of rectal patients, and overall rate of mesorectal excision was 96%. Rate of anastomotic leakage was 14%. |
| Elferink, M. A., et al., (2010) (49) | The Netherland | Rectal  2001-2006  16039  Cohort retrospective  Cancer registry | Yes | Rates of eligible treated according to guidelines (a / a+b) | Preoperative RT in T2-T3 M0  Chemoradiation in locally advanced |  | Overall rate of preoperatrive RT not explicitly reported (only on graphic); 70% in <75 years, 57% in >75 years.  Rate not explicitly reported (only on graphic) for chemoradiation in locally advanced.  Variation in treatment are documented.. |
| Elferink, M. A., et al., (2010) (50) | The Netherlands | Rectal  2001- 2006  11128  cross sectional  cancer registry + clinical records | Yes | Rates of eligible treated according to guidelines (a / a+b) | Rate of preoperative RT in pts with T2-T3 tumours, and of and chemoradiation in locally advanced tumours |  | Overall rates not explicitly reported (only on graphic). Variations in rates across hospitals |
| van Steenbergen, L. N., et al., (2010) (51) | The Netherlands | Colon  2001 – 2007  1637  Cohort retrospective  Cancer registry | yes | Rates of eligible treated according to guidelines (a / a+b) | Adjuvant chemotherapy in stage III |  | The proportion of patients receiving adjuvant chemotherapy decreased with increasing age from: 85% for patients <65 yr to 68% for those 65-74 yr and 17% for patients over75 yr, with large inter-hospital variation. Elderly patients and those with comorbidity received adjuvant chemotherapy less often. Patients with an intermediate or high socioeconomic status or stage IIIC received adjuvant chemotherapy more often. Undertreatment for elderly patients and wide inter-hospital variation is highlighted. |
| Hackl, C., M. et al., (2011) (52) | Germany | Colorectal (with liver metastates)  2002  236  cohort retrospective  cancer registry | No | Generic rate | Rate of liver resection |  | Rate of liver resection was 19.1%. rate was higher (40%) for the subgroup with single metastatic lesion . Underuse of curative liver resection is suggested. |
| Manchon-Walsh, P., et al., (2011) (53) | Spain | Rectal  2005 – 2007  1229  Cohort retrospective  Clinical records | yes | Rate of procedures performed according to guidelines (a/a+c) | Pre-operative RT / chemotherapy in Stage II – III patients |  | Overall, rate of pre-operative RT/chemotherapy was 54% |
| Manchon-Walsh, P., et al., (2011) (54) | Spain | Rectal  2005 – 2007  1831  Cohort retrospective  Clinical records | no | Generic | Total mesorectal excisions |  | Variation in surgical management across hospitals and overall low quality of clinical records |
| Mroczkowski P (2011) (55) | Poland | Rectal  2008 – 2009  709  Cohort prospective  Ad hoc data collection | No | Generic rates | Surgical procedures and pre-operative assessment |  | Patterns of care are presented as preliminary data, without drawing any conclusion |
| Quipourt, V., et al., (2011) (56) | French | Colorectal  2004-2007  2921  cohort retrospective  cancer register and clinical charts | Yes | Rates of eligible treated according to guidelines (a / a+b) | Curative surgical resection  Adjuvant chemotherapy in stage III Colon  Palliative chemotherapy  Radiotherapy for rectal cancer |  | Rates of curative resection were 66% in pts aged 75 or older, vs 75.8% for those younger.  40.4% of those aged 75 or older received chemotherapy in Stage III cancer, vs 90.5% for those younger.  Rates of palliative chemotherapy were 29% in >75 vs 77.1 in younger.  RT for rectal cancer was used in 59% of pts >75, vs 85.3% in those younger . Underuse, expecially of chemotherapy in stage III cancer and of palliative chemotherapy, not fully explained by comorbidities |
| Bonifazi, M., et al., (2012) (57) | Italy | Colorectal (metastatic)  2006-2007  637  cross-sectional  administrative databases | Yes | Rate of procedures performed according to guidelines (a/a+c) | Chemotherapy (bevacizumab) | 37% (34% - 42%) |  |
| Mathoulin-Pelissier, S., et al., (2012) (58) | France | Colorectal  2003-2004  825 colon  cohort prospective  clinical charts | Yes | Rates of eligible treated according to guidelines (a / a+b) | Use of adjuvant chemotherapy in stage II and in stage III |  | Overall rates of adjuvant chemotherapy were 26% and 71%, in stage II and stage III, respectively. Variation across centres was observed, and underuse of adjuvant chemo in stage III is highlighted. |
| Morris, S., G. et al., (2012) (59) | UK | Colon  2006 – 2009  24180  cohort retrospective  specialized database | Yes | Rates of eligible treated according to standard care (a / a+b) | Rate of colonscopy after positive occult blood test in adults aged 60-69 |  | Overall rate was 88%, with statistically significant variation between quintiles of area deprivation, as well as age and sex groups, ethnicity and geographical regions. |
| Khalid, U., et al., (2013) (60) | UK | Colorectal (T1 N0)  2009 – 2011  161 (112 colon, 49 rectal)  Cohort retrospective  Specialized database | Yes | Rates of not eligible exposed to treatments (C/C+D) | Major surgical resection vs local excision in T1 | 83% (77% - 89%) |  |
| Penninckx, F., et al., (2013) (61) | Belgium | Rectal  2006 – 2011  3197  Cross-sectional  Dedicated database | No | Generic rates | Abdominoperineal excision (AE) |  | Overall rate of AE was 21.1 %, with significant between centres variation, before and after adjustment for patient characteristics |
| Sjovall, A., et al., (2013) (62) | Sweden | Colon  2007 – 2010  15016  cohort retrospective  specialized database | Yes | Rates of eligible treated according to guidelines (a / a+b) | Abdomen CT/MRI and evaluation of lung and liver in staging |  | 65% of all patients had a complete radiological evaluation of the primary tumour, while 80% had examination of liver and lung . Overall, complete staging was executed in 60% of the patients. No staging of primary tumour was observed in 35% , and overall 39% of patients had incomplete staging evaluation. Variation among providers was observed. |
| Young AL (2013) (63) | UK | Colorectal  2009 – 2010  631  Cohort prospective  Ad hoc data collection | Yes | Rates of eligible treated according to guidelines (a / a+b) | Referral to surgery for patients with liver metastases |  | Variation in referral rates across hospitals. 142 patients referred to liver specialists, 107 treated with curative intent. A considerable number of patients with potentially curative liver metastases not referred to specialist surgeons |
| Van Leersum, N. J., et al., (2013) (64) | The Netherlands | Colorectal  2009 – 2011  26511  Cross sectional  Ad hoc data collection | Yes | Rates of eligible treated according to guidelines (a / a+b) | Colon : Total colonscopy;  Rectum : total colonscopy, pre-operative MR-imaging |  | Total colonscopy rates were 61% and 67% for colon cancer, in 2009 and 2011 respectively. Corresponding figures for rectal cancer were 76% and 83%.  Rates of MR pre-operative imaging in recatl cancer were 80% and 85%, in 2009 and 2011 respectively. |
| van Steenbergen, L. N., et al., (2013) (65) | The Netherlands | Colorectal  2003-2005 and 2008  450 in 2003-2005  129 2008  Cohort retrospective  Cancer registry + clinical records | Yes | Rates of eligible treated according to guidelines (a / a+b) | Colonscopy, control visits, ultrasound liver and CEA measurements in follow up |  | Overall, only 1% of patients were followed up according to the guidelines in the first year. Intensity of followed according to age and hospital. |
| Elliot, A. H., et al., (2014) (66) | Sweden | Rectal  2000-2010  2619  Cohort retrospective | yes | Rates of not eligible exposed to treatments (C/C+D) | Pre-operative RT (with or without chemotherapy) in early rectal cancer | 55% (50% - 60%) |  |
| Jannasch, O., et al., (2014) (67) | Germany  Poland  Italy | Colon  2009 – 2010  6.756 colon | No | Generic rates | Preoperative procedures  CT abdomen  CT thorax  Colonscopy  Tumour markers  Laparoscopic approach |  | Rates were  92.5% for CT abdomen; 50.9% for CT thorax; 86.3% for colonscopy; 71.7% for tumour markers; 15.8 for laparoscopic approach  Significant variation across the three counties |
|  |  | rectal  2009 – 2010  5935 rectal | No | Generic rates | Preoperative procedure  CT scan abdomen  MRI pelvis  Laparoscopic approach |  | Rates were  83.4% for CT abdomen; 31.2% for MRI pelvis; 16.6% for laparoscopic approach. Significant variation across the three counties |
| Lindskog, E. B., et al., (2014) (68) | Sweden | Colorectal stage in high risk II and III  2007-2012  10459  cohort retrospective  cancer registry | Yes | Rates of eligible treated according to guidelines (a / a+b) | Adjuvant chemotherapy in pts <76 year old |  | Rates of use of adjuvant chemotherapy in colon were 22% and 84%, in stage II and III, respectively. The corresponding figures for rectal cancer were 19% and 71%. Chemo not used in 42% and 45% of high risk stage II pts, for colon and rectal cancer respectively. Adherence to guidelines associated with younger age, presence of multidisciplinary teams, low-comoribity and worse N stage. |
| Minicozzi, P., et al., (2014) (69) | Italy, France | Rectal  2003-2005  1419  cohort retrospective  cancer registry+clinical documentation | Yes | Rates of eligible treated according to guidelines (a / a+b) | Surgery with curative intent; sphincter saving surger; preoperative radiotherapy in Stage II –III |  | Rate of surgery with curative intent was 69%, overall, without difference between the two countries. 21% of those undergoing curative surgery in Italy had a sphincter saving procedure, vs 32% in France.. Rate of preoperative radiotherapyin Stage II-III patients were 21% and 52%, in Italy and France respectively. |
| Rodriguez-Cuellar, E., (2014) (70) | Spain | Colorectal  2008  341  Cohort prospective  Ad hoc data collection | no | Rates of eligible treated according to guidelines (a / a+b) | Follow up procedures  Surgical management of recurrences  Adjuvant and neo-adjuvant chemotherapy |  | Only 19% were followed up by multidisciplinary teams. Only 61% of colon patients experiences recurrences had been exposed to adjuvant treatment. As for rectal cancer patients with recurrences, 44%, and 41% had neoadjuvant or adjuvant treatments, respectively. Increasing the rates of adoption of these treatments, of a multidisciplinary approach in follow-up, and of total mesenteric excision in rectal cancer are highlighted as areas of improvement. |
| Suchanek, S., et al., (71)(2014) | Czech Republic | Colorectal  2001 – 2010  NA  Cross sectional  Dedicated database | Yes | Rates of eligible treated according to guidelines (a / a+b) | Preventive colonscopy (ie. colonscopy after positive fecal occult blood test, or in individuals aged >55) |  | Rates ranging from 5.4% in 2001 to 22.7% in 2010, with significant variation both across geographic areas and age/sex population ‘s subgroups |
| Wesselmann, S., et al., (2014) (72) | Germany | Colorectal  2010 – 2012  19,567  Cross-sectional  Dedicated database | yes | Rates of eligible treated according to guidelines (a / a+b) | Adjuvant chemotherapy in colon  Neoadjuvant RT or chemo-radiotherapy in rectum |  | Median compliance rates were 73.7% and 80%, for adjuvant chemotherapy in colon cancer, and neoadjuvant Rt or chemoRT in rectum cancer, respectively |
| Herman, K. J., et al., (2015) (73) | Poland | Rectal  2005-2007  15.281  Cohort retrospective  Cancer registry | No | Generic rates | Rates of use of  RT  Chemo  Curative surgery |  | Rates were  47,5 % for RT ; 60,7 % for chemotherapy; 64,1 % for curative surgery  Significant variations across geographic regions |
| ‘t Lam Boer (2015) (74) | The Netherlands | Rectal  2004-2012  1617  Cohort retrospective  Cancer registry | No | Generic rates | Liver resection in stage IV patients with liver metastases |  | Large institutional variation is use of metatasectomy , which increased from 8% in 2004 to 24% in 2012 |

**Additional file 2c: Lung cancer**

|  | **Country** | **Years of study conduct, sample size, study design, source of information** | **Explicit standard** | **Measures** | **Interventions/procedures** | **Prevalence of overuse (95%CI)** | **Conclusions** |
| --- | --- | --- | --- | --- | --- | --- | --- |
| Patel, N., et al., (2007) (75) | UK | 1994-2003  NSCLC 31977  SCLC 8668  Not specified 2667  Cross-sectional  Cancer registry | No | Generic rates | Rate of use of chemotherapy |  | Increase in use of chemotherapy (from 13,6% to 29,3) over the study period. Variation in use related to age, stage of disease, type of lung cancer, geographic area. |
| Li WWL , et al (2008) (76) | The Netherlands | 1998 – 2003  NSCLC 1591  Cohort retrospective  Cancer registry | No | Generic rates | Surgical resection |  | &9% patients had surgery with significant variation across hospitals (from 48% to 90%). Patients diagnosed at specialised centres or higher volume hospitals more likely to receive surgical therapy. |
| Wouters, M. W., et al., (2010) (77) | The Netherlands | 2001-2006  43544  cross-sectional  cancer registry + clinical records | Yes | Rate of eligible treated according to guidelines ( a / a+b) | Resection rate in Stage I-II  Chemoradiation for stage III  Chemotherapy in stage IV |  | 60% for resection rate in Stage I-II  29% for chemoradiation in stage III  50%for chemotherapy in Stage IV  Observed variations across hospitals and geographic areas |
| Grose, D., et al., (2011) (78) | Scotland | 2005 – 2008  882 (62% NSCLC)  Cohort prospective  Ad hoc data collection | Yes | Rates of eligible treated according to guidelines (a / a+b) | Surgical resection;  radical radiotherapy  Chemotherapy in NSCLC stage IIIb or IV  Supportive care |  | Rates were 6.3% for surgical resection; 4.0% for radical radiotherapy; 10.7% for chemotherapy in Stage IIIb-IV NSCLC; 20.6% for supportive care. Significant between centres variation was documented for the last three items of care. |
| Rich, A. L., et al., (2011)(79) | UK | 2004 – 2007  60,059  Cross-sectional  Specialized database | No | Generic rates | Surgical treatment;  Chemotherapy  Radiotherapy |  | Rates of use of surgery, chemotherapy and radiotherapy assessed according to patient characteristics. Little variation emerged as for patient socio-economic status |
| Riaz, S. P., et al., (2012) (80) | UK | 2004 – 2006  77349  cohort retrospective  cancer registry + administrative databases | No | Generic rates | Rate of surgical resections |  | Overall rate was 9%, with large geographical variation. |
| Lau, K. K., et al., (2013) (81) | UK | 2009  25029 (17948 NSCLC)  Cross-sectional  Specialized databases | Yes | Generic rates | Surgical resection in NSCLC |  | Overall rate of surgical resection was 18%. Large variation in the resection rate was seen, partially related to the local availability of specialist thoracic surgeons |
| Tovar, I., et al., (2013) (82) | Spain | 2007  3051  cohort retrospective  cancer registry+ clinical records | Yes | Rates of eligible treated according to guidelines (a / a+b) | Rate of radiotherapy |  | Overall rate was 20%, Estimated underuse was 25%. |

**Additional file 2d: Prostate cancer**

|  | **Country** | **Years of study conduct, sample size, study design, source of information** | **Explicit standard** | **Measures** | **Interventions/procedures** | **Prevalence of overuse (95%CI)** | **Conclusions** |
| --- | --- | --- | --- | --- | --- | --- | --- |
| Hernes, E., et al., (2010) (83) | Norway | 2004  1650  Cohort retrospective  Administrative database | Yes | Rates of not eligible exposed to treatments (C/C+D) | Radical prostatectomy or Radiotherapy in low risk patients | 57% ( (52% - 61%) | . |
| Drummond, F. J., et al., (2010) (84) | Ireland | 1995 – 2005  NA  Cross-sectional  Ad hoc data collection | No | Generic rates | PSA |  | Age-standardised rates of PSA tests performed increased by 26% yearly study period |
| Evans, S., et al., (2010) (85) | UK | 2001 (?)  1866  cohort retrospective  cancer registry+clinical records | Yes | Rates of not eligible exposed to treatments (C/C+D) | CT scan  radical prostatectomy  radiotherapy  hormone therapy | 10% (7% - 11%)  < 1%  < 1%  < 1% |  |
| Lyratzopoulos, G., et al., (2010) (86) | UK | 1995-2006  35171  cross-sectional | No | Generic rates | Rate of radiotherapy and of radical prostatectomy |  | 25.3% for radiotherapy and 6.7% for radical prostatectomy over the study period. Variation in rates across socioeconomic groups , with lower rates in pts with lower socioeconomic status |
| Grundmark, B., et al., (2012) (87) | Sweden | 1997 – 2006  2560  Cohort retrospective  Cancer registry | Yes | Rate of procedures performed according to guidelines (a/a+c) | Anti-androgen (bicalutamide) in low/intermediate risk patients | 2.1% (? - ?) |  |
| Hamoen, E. H., et al., (2013) (88) | The Netherlands | 2002-2011  >65000 male patients  cohort retrospective  administrative databases | No | Generic rates | Rates of PSA testing |  | Incidence of PSA testing in men aged >45 years increased from 15.5 per 1000 person-years in 2002 to 54.3 per 1000 person-years in 2011. In those over 75 years, rates were 29.2 per 1000 person years in 2002 and 96.7 per 1000 person years in 2011. |
| Bratt, O., et al., (2015) (89) | Sweden | 2001-2012  19190  cross-sectional  specialised database | Yes | Rates of eligible treated according to guidelines (a / a+b) | Rate of use of radical prostatectomy, radiotherapy |  | Overall rates were 16.9% for radical prostatectomy and 28.8 for radiotherapy. Underuse of curative treatments in the elderly, not related to their life expectancy. |
| Hager, B., et al., (2015) (90) | Germany | 2004 – 2011  54159  Cross-sectional  Cancer registry | No | Generic rates | Radical prostatectomy, radiotherapy, no active treatment; |  | Rates were 66% for radical prostatectomy, and 11,8% for radiotherapy. No active treatment was adopted in 16%. The study provides a comparison with the USA, where use of radiotherapy appeared to be much higher (34%), with less frequent use of radical prostatectomy (36%). |
| Sachdeva, A., et al., (2015) (91) | UK (vs USA) | 2004 – 2008  25235 (UK)  196928 (USA)  Cohort retrospective  Cancer registry+administrative database | No | Generic rates | Definitive treatment (radical prostatectomy or RT) |  | Overall rate of curative primary treatment were 38% % (UK) vs 77% (USA). |

**Additional file 2e: Miscellanea (i.e. including different types of cancer)**

|  | **Country** | **Tumour, years of study conduct, sample size, study design, source of information** | **Explicit standard** | **Measures** | **Interventions/procedures** | **Prevalence of overuse (95%CI)** | **Notes** |
| --- | --- | --- | --- | --- | --- | --- | --- |
| Olasolo, J., et al., (2007) (92) | Spain | Various (including breast and lung)  2006  NA  Cross-sectional  Administrative database | No | Generic rates | Radiotherapy |  | Comparison of observed vs expected rates of use of radiotherapy highlighted possible underuse in lung cancer patients. Overall observed rates for breast cancer were in line with the expected one, but with substantial variation across provinces. |
| Vulto JCM, et al (2008) (93) | The Netherlands | Various including breast and rectal  2003 – 2006  19098  Cross-sectional  Cancer registry | No | Generic rates | Radiotherapy |  | Proportion of patients receiving radiotherapy remained stable over the study period. Geographic variation in rates were observed. |
| Apolone, G., et al., (2009) (94) | Italy | Various including breast, lung, and colorectal  2006-2007  1801  Cohort Prospective  Ad hoc data collection | Yes | Rates of eligible treated according to guidelines (a / a+b) | Analgesic drugs |  | Potential undertreatment in 25.3% of patients |
| Wojtukiewicz, M. Z., et al., (2009) (95) | Poland | Various,  2004  999 including lung (142), colorectal, breast (208) Colorectal (165)  cohort retrospective  ad hoc data collection | No | Rates of eligible treated according to guidelines (a / a+b) | Management of anemia |  | Incidence of anemia was 31% . Only 32% of pts undergoing chemotherapy and experiencing anemia received treatment for this condition. Anemia underestimated and undertreated. |
| Ray-Coquard, I., et al., (2012) (96) | France | Breast and lung (advanced)  2010  185 Breast 227 lung  cross-sectional  ad-hoc data collection | Yes | Rate of procedures performed according to guidelines (a/a+c) | Erythropoiesis stimulating agents in chemotherapy induced anemia | Overall prevalence of overuse 5% in breast (n=185) and lung (n=227) cancer patients (20/ 412) |  |
| Singer, S., et al., (2013) (97) | Germany | Various  2009  36165  cross-sectional  ad-hoc data collection | Yes | Generic rate | Psycho-oncological care |  | 37% patients received psycho-oncological care- no explicit mention of problems of over or underuse |
| Joerger, M., et al., (2014) (98) | Switzerland | Various including breast, lung, and colorectal  2012  354  Cohort Prospective  Ad hoc data collection | Yes | Rate of procedures performed according to guidelines (a/a+c) | Anticancer drugs | Results by cancer site reported only in graphic. Overall, 32% of all patients received at least one-off label drug, but off label use was unsupported by European Society for Medical Oncology guidelines only in 6.6% of cases. Inappropriate use was higher for bevacizumab (29%) also because of its use in advanced breast cancer |  |
| Sevestre, M. A., et al., (2014) (99) | France | Various  2010 – 2011  500  cross-sectional  ad hoc data collection | Yes | Rates of eligible treated according to guidelines(a / a+b) | Management of venous thromboembolism with at least three months of low molecular weight heparine (LMWH) |  | Overall compliance was 59%. Underuse of LMWH |
| Vogt, V., et al., (2014) (100) | Germany | Breast, prostate, colorectal  2008 – 2011  NA  Cross-sectional | No | Generic rates | Uptake of screening tests  mammography  colonscopy,  PSA |  | Overall, rates were 19% for mammography, 1.7% for colonscopy, and 21% for PSA. Substantial variation was observed, and rates found significantly higher in areas with higher physician density. |

Bibliography

1. Fedeli U, Alba N, Schievano E, Visentin C, Rosato R, Zorzi M, et al. Diffusion of good practices of care and decline of the association with case volume: the example of breast conserving surgery. BMC Health Serv Res. 2007;7:167.

2. Ponti A, Mano MP, Distante V, Baiocchi D, Bordon R, Federici A, et al. Audit system on quality of breast cancer diagnosis and treatment (QT): results from the survey on screen-detected lesions in Italy, 2004. Epidemiol Prev. 2007;31(2-3 Suppl 2):69-75.

3. Ponti A, Mano MP, Tomatis M, Baiocchi D, Barca A, Berti R, et al. Audit system on quality of breast cancer diagnosis and treatment (QU): results of quality indicators on screen-detected lesions in Italy, 2011-2012. Epidemiol Prev. 2015;39(3):40-7.

4. Ponti A, Tomatis M, Baiocchi D, Barca A, Berti R, Bisanti L, et al. Audit on quality of breast cancer diagnosis and treatment in Italy, 2008-2009. Epidemiol Prev. 2011;35(5-6 Suppl 5):87-95.

5. Brucker SY, Schumacher C, Sohn C, Rezai M, Bamberg M, Wallwiener D. Benchmarking the quality of breast cancer care in a nationwide voluntary system: the first five-year results (2003-2007) from Germany as a proof of concept. BMC Cancer. 2008;8:358.

6. Brucker SY, Wallwiener M, Kreienberg R, Jonat W, Beckmann MW, Bamberg M, et al. Optimizing the quality of breast cancer care at certified german breast centers: a benchmarking analysis for 2003-2009 with a particular focus on the interdisciplinary specialty of radiation oncology. Strahlenther Onkol. 2011;187(2):89-99.

7. Poncet B, Colin C, Bachelot T, Jaisson-Hot I, Derain L, Magaud L, et al. Treatment of metastatic breast cancer: a large observational study on adherence to French prescribing guidelines and financial cost of the anti-HER2 antibody trastuzumab. Am J Clin Oncol. 2009;32(4):369-74.

8. Rosato R, Sacerdote C, Pagano E, Di Cuonzo D, Baldi I, Bordon R, et al. Appropriateness of early breast cancer management in relation to patient and hospital characteristics: a population based study in Northern Italy. Breast Cancer Res Treat. 2009;117(2):349-56.

9. Allemani C, Storm H, Voogd AC, Holli K, Izarzugaza I, Torrella-Ramos A, et al. Variation in 'standard care' for breast cancer across Europe: a EUROCARE-3 high resolution study. Eur J Cancer. 2010;46(9):1528-36.

10. Ess S, Savidan A, Frick H, Rageth C, Vlastos G, Lutolf U, et al. Geographic variation in breast cancer care in Switzerland. Cancer Epidemiol. 2010;34(2):116-21.

11. Jeevan R, Cromwell DA, Browne JP, Trivella M, Pereira J, Caddy CM, et al. Regional variation in use of immediate breast reconstruction after mastectomy for breast cancer in England. Eur J Surg Oncol. 2010;36(8):750-5.

12. Mano MP, Ponti A, Tomatis M, Baiocchi D, Barca A, Berti R, et al. Audit system on Quality of breast cancer diagnosis and Treatment (QT): results of quality indicators on screen-detected lesions in Italy, 2007. Epidemiol Prev. 2010;34(5-6 Suppl 4):81-8.

13. Montemurro F, Rossi V, Nole F, Redana S, Donadio M, Martinello R, et al. Underuse of anthracyclines in women with HER-2+ advanced breast cancer. Oncologist. 2010;15(7):665-72.

14. van Steenbergen LN, van de Poll-Franse LV, Wouters MW, Jansen-Landheer ML, Coebergh JW, Struikmans H, et al. Variation in management of early breast cancer in the Netherlands, 2003-2006. Eur J Surg Oncol. 2010;36 Suppl 1:S36-43.

15. Wockel A, Kurzeder C, Geyer V, Novasphenny I, Wolters R, Wischnewsky M, et al. Effects of guideline adherence in primary breast cancer--a 5-year multi-center cohort study of 3976 patients. Breast. 2010;19(2):120-7.

16. Bastiaannet E, Portielje JE, van de Velde CJ, de Craen AJ, van der Velde S, Kuppen PJ, et al. Lack of survival gain for elderly women with breast cancer. Oncologist. 2011;16(4):415-23.

17. Lebeau M, Mathoulin-Pelissier S, Bellera C, Tunon-de-Lara C, Daban A, Lipinski F, et al. Breast cancer care compared with clinical guidelines: an observational study in France. BMC Public Health. 2011;11:45.

18. Lu W, Jansen L, Schaapveld M, Baas PC, Wiggers T, De Bock GH. Underuse of long-term routine hospital follow-up care in patients with a history of breast cancer? BMC Cancer. 2011;11(1):279.

19. Struikmans H, Aarts MJ, Jobsen JJ, Koning CC, Merkus JW, Lybeert ML, et al. An increased utilisation rate and better compliance to guidelines for primary radiotherapy for breast cancer from 1997 till 2008: a population-based study in the Netherlands. Radiother Oncol. 2011;100(2):320-5.

20. Weggelaar I, Aben KK, Warle MC, Strobbe LJ, van Spronsen DJ. Declined guideline adherence in older breast cancer patients: a population-based study in the Netherlands. Breast J. 2011;17(3):239-45.

21. Clavarezza M, Mustacchi G, Casadei Gardini A, Del Mastro L, De Matteis A, Riccardi F, et al. Biological characterization and selection criteria of adjuvant chemotherapy for early breast cancer: experience from the Italian observational NEMESI study. BMC Cancer. 2012;12(1):216.

22. Fong A, Shafiq J, Saunders C, Thompson AM, Tyldesley S, Olivotto IA, et al. A comparison of surgical and radiotherapy breast cancer therapy utilization in Canada (British Columbia), Scotland (Dundee), and Australia (Western Australia) with models of "optimal" therapy. Breast. 2012;21(4):570-7.

23. Grandjean I, Kwast AB, de Vries H, Klaase J, Schoevers WJ, Siesling S. Evaluation of the adherence to follow-up care guidelines for women with breast cancer. Eur J Oncol Nurs. 2012;16(3):281-5.

24. Schwentner L, Wockel A, Konig J, Janni W, Ebner F, Blettner M, et al. Adherence to treatment guidelines and survival in triple-negative breast cancer: a retrospective multi-center cohort study with 9,156 patients. BMC Cancer. 2013;13:487.

25. Schwentner L, Wolters R, Koretz K, Wischnewsky MB, Kreienberg R, Rottscholl R, et al. Triple-negative breast cancer: the impact of guideline-adherent adjuvant treatment on survival--a retrospective multi-centre cohort study. Breast Cancer Res Treat. 2012;132(3):1073-80.

26. Schwentner L, Wolters R, Wischnewsky M, Kreienberg R, Wockel A. Survival of patients with bilateral versus unilateral breast cancer and impact of guideline adherent adjuvant treatment: a multi-centre cohort study of 5292 patients. Breast. 2012;21(2):171-7.

27. Stordeur S, Vrijens F, Devriese S, Beirens K, Van Eycken E, Vlayen J. Developing and measuring a set of process and outcome indicators for breast cancer. Breast. 2012;21(3):253-60.

28. van de Water W, Bastiaannet E, Dekkers OM, de Craen AJ, Westendorp RG, Voogd AC, et al. Adherence to treatment guidelines and survival in patients with early-stage breast cancer by age at diagnosis. Br J Surg. 2012;99(6):813-20.

29. Vrijens F, Stordeur S, Beirens K, Devriese S, Van Eycken E, Vlayen J. Effect of hospital volume on processes of care and 5-year survival after breast cancer: a population-based study on 25000 women. Breast. 2012;21(3):261-6.

30. Joerger M, Thurlimann B, Savidan A, Frick H, Rageth C, Lutolf U, et al. Treatment of breast cancer in the elderly: a prospective, population-based Swiss study. J Geriatr Oncol. 2013;4(1):39-47.

31. Leoni M, Sadacharan R, Louis D, Falcini F, Rabinowitz C, Cisbani L, et al. Variation among local health units in follow-up care of breast cancer patients in Emilia-Romagna, Italy. Tumori. 2013;99(1):30-4.

32. Arican A, Bozkurt T, Bozcuk H, Demirkan B, Buyukberber S, Alkis N, et al. A cross-sectional survey of the diagnosis and management of bone metastasis in breast cancer patients in Turkey. Support Care Cancer. 2014;22(10):2629-34.

33. Gion M, Franceschini R, Rosin C, Trevisiol C, Peloso L, Zappa M, et al. An epidemiology-based model to estimate the rate of inappropriateness of tumor marker requests. Clin Chem Lab Med. 2014;52(6):889-97.

34. Gion M, Peloso L, Trevisiol C, Squarcina E, Zappa M, Fabricio AS. An epidemiology-based model as a tool to monitor the outbreak of inappropriateness in tumor marker requests: a national scale study. Clin Chem Lab Med. 2016;54(3):473-82.

35. Ponti A, Lynge E, James T, Majek O, von Euler-Chelpin M, Anttila A, et al. International variation in management of screen-detected ductal carcinoma in situ of the breast. Eur J Cancer. 2014;50(15):2695-704.

36. Dialla PO, Quipourt V, Gentil J, Marilier S, Poillot ML, Roignot P, et al. In breast cancer, are treatments and survival the same whatever a patient's age? A population-based study over the period 1998-2009. Geriatr Gerontol Int. 2015;15(5):617-26.

37. Inwald EC, Klinkhammer-Schalke M, Koller M, Ortmann O. Quality Assurance for Patients with Breast Cancer – the Impact of Clinical Cancer Registries. Geburtshilfe und Frauenheilkunde. 2014;74(9):868-74.

38. Kelly E, Lu CY, Albertini S, Vitry A. Longitudinal trends in utilization of endocrine therapies for breast cancer: an international comparison. J Clin Pharm Ther. 2015;40(1):76-82.

39. Kiderlen M, Ponti A, Tomatis M, Boelens PG, Bastiaannet E, Wilson R, et al. Variations in compliance to quality indicators by age for 41,871 breast cancer patients across Europe: a European Society of Breast Cancer Specialists database analysis. Eur J Cancer. 2015;51(10):1221-30.

40. Massat NJ, Douglas E, Waller J, Wardle J, Duffy SW. Variation in cervical and breast cancer screening coverage in England: a cross-sectional analysis to characterise districts with atypical behaviour2015.

41. Morgan J, Richards P, Ward S, Francis M, Lawrence G, Collins K, et al. Case-mix analysis and variation in rates of non-surgical treatment of older women with operable breast cancer. Br J Surg. 2015;102(9):1056-63.

42. Gondos A, Jansen L, Heil J, Schneeweiss A, Voogd AC, Frisell J, et al. Time trends in axilla management among early breast cancer patients: Persisting major variation in clinical practice across European centers. Acta Oncol. 2016;55(6):712-9.

43. Richards P, Ward S, Morgan J, Lagord C, Reed M, Collins K, et al. The use of surgery in the treatment of ER+ early stage breast cancer in England: Variation by time, age and patient characteristics. Eur J Surg Oncol. 2016;42(4):489-96.

44. Rococo E, Mazouni C, Or Z, Mobillion V, Koon Sun Pat M, Bonastre J. Variation in rates of breast cancer surgery: A national analysis based on French Hospital Episode Statistics. Eur J Surg Oncol. 2016;42(1):51-8.

45. Lepage C, Bouvier AM, Binquet C, Dancourt V, Coatmeur O, Faivre J. Are the recommendations of the French consensus conference on the management of colon cancer followed up? Eur J Cancer Prev. 2006;15(4):295-300.

46. Adler A, Roll S, Marowski B, Drossel R, Rehs HU, Willich SN, et al. Appropriateness of colonoscopy in the era of colorectal cancer screening: a prospective, multicenter study in a private-practice setting (Berlin Colonoscopy Project 1, BECOP 1). Dis Colon Rectum. 2007;50(10):1628-38.

47. Morris E, Quirke P, Thomas JD, Fairley L, Cottier B, Forman D. Unacceptable variation in abdominoperineal excision rates for rectal cancer: time to intervene? Gut. 2008;57(12):1690-7.

48. Kube R, Ptok H, Wolff S, Lippert H, Gastinger I. Quality of medical care in colorectal cancer in Germany. Onkologie. 2009;32(1-2):25-9.

49. Elferink MA, Wouters MW, Krijnen P, Lemmens VE, Jansen-Landheer ML, van de Velde CJ, et al. Disparities in quality of care for colon cancer between hospitals in the Netherlands. Eur J Surg Oncol. 2010;36 Suppl 1:S64-73.

50. Elferink MA, Krijnen P, Wouters MW, Lemmens VE, Jansen-Landheer ML, van de Velde CJ, et al. Variation in treatment and outcome of patients with rectal cancer by region, hospital type and volume in the Netherlands. Eur J Surg Oncol. 2010;36 Suppl 1:S74-82.

51. van Steenbergen LN, Rutten HJ, Creemers GJ, Pruijt JF, Coebergh JW, Lemmens VE. Large age and hospital-dependent variation in administration of adjuvant chemotherapy for stage III colon cancer in southern Netherlands. Ann Oncol. 2010;21(6):1273-8.

52. Hackl C, Gerken M, Loss M, Klinkhammer-Schalke M, Piso P, Schlitt HJ. A population-based analysis on the rate and surgical management of colorectal liver metastases in Southern Germany. Int J Colorectal Dis. 2011;26(11):1475-81.

53. Manchon-Walsh P, Borras JM, Espinas JA, Aliste L. Assessing the effectiveness of a guideline recommendation for pre-operative radiochemotherapy in rectal cancer. Radiother Oncol. 2011;99(2):142-7.

54. Manchon Walsh P, Borras JM, Espinas JA, Aliste L. Variability in the quality of rectal cancer care in public hospitals in catalonia (Spain): clinical audit as a basis for action. Eur J Surg Oncol. 2011;37:325-33.

55. Mroczkowski P, Hac S, Mik M, Berut M, Dziki L, Kube R, et al. Preliminary results of the first quality assurance project in rectal cancer in Poland. Pol Przegl Chir. 2011;83(3):144-9.

56. Quipourt V, Jooste V, Cottet V, Faivre J, Bouvier AM. Comorbidities alone do not explain the undertreatment of colorectal cancer in older adults: a French population-based study. J Am Geriatr Soc. 2011;59(4):694-8.

57. Bonifazi M, Rossi M, Moja L, Scigliano VD, Franchi M, La Vecchia C, et al. Bevacizumab in clinical practice: prescribing appropriateness relative to national indications and safety. Oncologist. 2012;17(1):117-24.

58. Mathoulin-Pelissier S, Becouarn Y, Belleannee G, Pinon E, Jaffre A, Coureau G, et al. Quality indicators for colorectal cancer surgery and care according to patient-, tumor-, and hospital-related factors. BMC Cancer. 2012;12:297.

59. Morris S, Baio G, Kendall E, von Wagner C, Wardle J, Atkin W, et al. Socioeconomic variation in uptake of colonoscopy following a positive faecal occult blood test result: a retrospective analysis of the NHS Bowel Cancer Screening Programme. Br J Cancer. 2012;107(5):765-71.

60. Khalid U, Evans MD, Williams GL, Hanson J, Davies M. Variability in management of T1 colorectal cancer in Wales. Ann R Coll Surg Engl. 2013;95(7):477-80.

61. Penninckx F, Fieuws S, Beirens K, Demetter P, Ceelen W, Kartheuser A, et al. Risk adjusted benchmarking of abdominoperineal excision for rectal adenocarcinoma in the context of the Belgian PROCARE improvement project. Gut. 2013;62(7):1005-11.

62. Sjovall A, Blomqvist L, Martling A. Pretreatment staging of colon cancer in the Swedish population. Colorectal Dis. 2013;15(11):1361-6.

63. Young A, Adair R, Culverwell A, Guthrie J, Botterill I, Toogood G, et al. Variation in referral practice for patients with colorectal cancer liver metastases. Br J Surg. 2013;100:1627 - 32.

64. Van Leersum NJ, Snijders HS, Henneman D, Kolfschoten NE, Gooiker GA, ten Berge MG, et al. The Dutch surgical colorectal audit. Eur J Surg Oncol. 2013;39(10):1063-70.

65. van Steenbergen LN, de Hingh IH, Rutten HJ, Rijk MC, Orsini RG, Coebergh JW, et al. Large variation between hospitals in follow-up for colorectal cancer in southern Netherlands. Int J Colorectal Dis. 2013;28(9):1257-65.

66. Elliot AH, Martling A, Glimelius B, Nordenvall C, Johansson H, Nilsson PJ. Preoperative treatment selection in rectal cancer: a population-based cohort study. Eur J Surg Oncol. 2014;40(12):1782-8.

67. Jannasch O, Udelnow A, Romano G, Dziki A, Pavalkis D, Lippert H, et al. International quality assurance project in colorectal cancer-unifying diagnostic and histopathological evaluation. Langenbecks Arch Surg. 2014;399(4):473-9.

68. Lindskog EB, Gunnarsdottir KA, Derwinger K, Wettergren Y, Glimelius B, Kodeda K. A population-based cohort study on adherence to practice guidelines for adjuvant chemotherapy in colorectal cancer. BMC Cancer. 2014;14:948.

69. Minicozzi P, Bouvier AM, Faivre J, Sant M. Management of rectal cancers in relation to treatment guidelines: a population-based study comparing Italian and French patients. Dig Liver Dis. 2014;46(7):645-51.

70. Rodriguez-Cuellar E, Nevado Garcia C, Casanova Duran V, Romero Simo M, Duran Poveda M, Ruiz Lopez P. Analysis of the quality of care in surgical treatment of colorectal cancer: national study. Follow-up results. Cir Esp. 2014;92(6):410-4.

71. Suchanek S, Majek O, Vojtechova G, Minarikova P, Rotnaglova B, Seifert B, et al. Colorectal cancer prevention in the Czech Republic: time trends in performance indicators and current situation after 10 years of screening. Eur J Cancer Prev. 2014;23(1):18-26.

72. Wesselmann S, Winter A, Ferencz J, Seufferlein T, Post S. Documented quality of care in certified colorectal cancer centers in Germany: German Cancer Society benchmarking report for 2013. Int J Colorectal Dis. 2014;29(4):511-8.

73. Herman KJ, Komorowski AL, Wysocki WM, Tabor J, Herman RM, Sliwczynski A. Variation in treatment modalities, costs and outcomes of rectal cancer patients in Poland. Contemp Oncol 2015;19(5):400-9.

74. 't Lam-Boer J, Al Ali C, Verhoeven R, Roumen R, Lemmens V, Rijken A, et al. Large variation in the utilization of liver resections in stage IV colorectal cancer patients with metastases confined to the liver. Eur J Cancer Surg. 2015;41:1217-25.

75. Patel N, Adatia R, Mellemgaard A, Jack R, Moller H. Variation in the use of chemotherapy in lung cancer. Br J Cancer. 2007;96(6):886-90.

76. Li WW, Visser O, Ubbink DT, Klomp HM, Kloek JJ, de Mol BA. The influence of provider characteristics on resection rates and survival in patients with localized non-small cell lung cancer. Lung Cancer. 2008;60(3):441-51.

77. Wouters MW, Siesling S, Jansen-Landheer ML, Elferink MA, Belderbos J, Coebergh JW, et al. Variation in treatment and outcome in patients with non-small cell lung cancer by region, hospital type and volume in the Netherlands. Eur J Surg Oncol. 2010;36 Suppl 1:S83-92.

78. Grose D, Devereux G, Brown L, Jones R, Sharma D, Selby C, et al. Variation in comorbidity and clinical management in patients newly diagnosed with lung cancer in four Scottish centers. J Thorac Oncol. 2011;6(3):500-9.

79. Rich AL, Tata LJ, Stanley RA, Free CM, Peake MD, Baldwin DR, et al. Lung cancer in England: information from the National Lung Cancer Audit (LUCADA). Lung Cancer. 2011;72(1):16-22.

80. Riaz SP, Luchtenborg M, Jack RH, Coupland VH, Linklater KM, Peake MD, et al. Variation in surgical resection for lung cancer in relation to survival: population-based study in England 2004-2006. Eur J Cancer. 2012;48(1):54-60.

81. Lau KK, Rathinam S, Waller DA, Peake MD. The effects of increased provision of thoracic surgical specialists on the variation in lung cancer resection rate in England. J Thorac Oncol. 2013;8(1):68-72.

82. Tovar I, Exposito J, Jaen J, Alonso E. Underuse of radiotherapy in lung cancer has negative consequences for patients. J Thorac Oncol. 2013;8(1):62-7.

83. Hernes E, Kyrdalen A, Kvale R, Hem E, Klepp O, Axcrona K, et al. Initial management of prostate cancer: first year experience with the Norwegian National Prostate Cancer Registry. BJU Int. 2010;105(6):805-11; discussion 11.

84. Drummond FJ, Carsin AE, Sharp L, Comber H. Trends in prostate specific antigen testing in Ireland: lessons from a country without guidelines. Ir J Med Sci. 2010;179(1):43-9.

85. Evans S, Metcalfe C, Patel B, Ibrahim F, Anson K, Chinegwundoh F, et al. Clinical presentation and initial management of black men and white men with prostate cancer in the United Kingdom: the PROCESS cohort study. Br J Cancer. 2010;102(2):249-54.

86. Lyratzopoulos G, Barbiere JM, Greenberg DC, Wright KA, Neal DE. Population based time trends and socioeconomic variation in use of radiotherapy and radical surgery for prostate cancer in a UK region: continuous survey. Bmj. 2010;340:c1928.

87. Grundmark B, Garmo H, Zethelius B, Stattin P, Lambe M, Holmberg L. Anti-androgen prescribing patterns, patient treatment adherence and influencing factors; results from the nationwide PCBaSe Sweden. Eur J Clin Pharmacol. 2012;68(12):1619-30.

88. Hamoen EH, Reukers DF, Numans ME, Barentsz JO, Witjes JA, Rovers MM. Discrepancies between guidelines and clinical practice regarding prostate-specific antigen testing. Fam Pract. 2013;30(6):648-54.

89. Bratt O, Folkvaljon Y, Hjalm Eriksson M, Akre O, Carlsson S, Drevin L, et al. Undertreatment of men in their seventies with high-risk nonmetastatic prostate cancer. Eur Urol. 2015;68(1):53-8.

90. Hager B, Kraywinkel K, Keck B, Katalinic A, Meyer M, Zeissig SR, et al. Integrated prostate cancer centers might cause an overutilization of radiotherapy for low-risk prostate cancer: A comparison of treatment trends in the United States and Germany from 2004 to 2011. Radiother Oncol. 2015;115(1):90-5.

91. Sachdeva A, van der Meulen JH, Emberton M, Cathcart PJ. Evaluating variation in use of definitive therapy and risk-adjusted prostate cancer mortality in England and the USA. BMJ Open. 2015;5(2):e006805.

92. Olasolo J, Alonso Redondo E, Exposito Hernandez J, de las Penas Cabrera MD, Cabrera Roldan P. Evidence-based estimation and radiotherapy utilisation rate in Andalusia. Clin Transl Oncol. 2007;9(12):789-96.

93. Vulto JC, Lybeert ML, Louwman MW, Poortmans PM, Coebergh JW. Population-based study of trends and variations in radiotherapy as part of primary treatment of cancer in the southern Netherlands between 1988 and 2006, with an emphasis on breast and rectal cancer. Int J Radiat Oncol Biol Phys. 2009;74(2):464-71.

94. Apolone G, Corli O, Caraceni A, Negri E, Deandrea S, Montanari M, et al. Pattern and quality of care of cancer pain management. Results from the Cancer Pain Outcome Research Study Group. Br J Cancer. 2009;100(10):1566-74.

95. Wojtukiewicz MZ, Sierko E, Rybaltowski M, Filipczyk-Cisarz E, Staroslawska E, Tujakowski J, et al. The Polish Cancer Anemia Survey (POLCAS): a retrospective multicenter study of 999 cases. Int J Hematol. 2009;89(3):276-84.

96. Ray-Coquard I, Morere JF, Scotte F, Cals L, Antoine EC. Management of anemia in advanced breast and lung cancer patients in daily practice: results of a French survey. Adv Ther. 2012;29(2):124-33.

97. Singer S, Dieng S, Wesselmann S. Psycho-oncological care in certified cancer centres--a nationwide analysis in Germany. Psycho-Oncology. 2013;22(6):1435-7.

98. Joerger M, Schaer-Thuer C, Koeberle D, Matter-Walstra K, Gibbons-Marsico J, Diem S, et al. Off-label use of anticancer drugs in eastern Switzerland: a population-based prospective cohort study. Eur J Clin Pharmacol. 2014;70(6):719-25.

99. Sevestre MA, Belizna C, Durant C, Bosson JL, Vedrine L, Cajfinger F, et al. Compliance with recommendations of clinical practice in the management of venous thromboembolism in cancer: the CARMEN study. J Mal Vasc. 2014;39(3):161-8.

100. Vogt V, Siegel M, Sundmacher L. Examining regional variation in the use of cancer screening in Germany. Soc Sci Med. 2014;110:74-80.
